# Supplementary material for: Body orientation change of neighbors leads to scale-free correlation in collective motion
Source: Nat Commun. 2024 Oct 17;15:8968. doi: 10.1038/s41467-024-53361-8 (PMC11487077; doi:10.1038/s41467-024-53361-8)
Supplement: Supplementary file 1 — Supplementary Information [file 41467_2024_53361_MOESM1_ESM.pdf]

Body Orientation Change of Neighbors Leads to Scale-Free Correlation in Collective Motion: Supplementary Information

Contents

Supplementary Notes **3**

1 Data processing of U-turn datasets **3**

2 Analysis of Experimental Data from U-turn behaviors **3**
- 2.1 Segmentation of U-turn trajectory . . . 3
- 2.2 Effectiveness of Leadership Evaluation Indicator . . . 3
- 2.3 Spatial Distribution of Turning Order in Fish Schools . . . 4
- 2.4 Distance-based motion salience . . . 4
- 2.5 Bearing Change-based motion salience . . . 4

3 Evaluation Indicators **5**
- 3.1 Direction of information transfer . . . 5
- 3.2 Polarization . . . 5

4 Reconstruct the visual field and involvement of visual occlusion from first-person perspective **5**

5 Definition of Baseline models **6**
- 5.1 Vicsek Model . . . 6

6 Details of swarm robotic validation system **6**
- 6.1 Architecture and workflow of swarm robotic validation system . . . 6
- 6.2 Simulate the visual sensing by the pinhole camera model . . . 7
- 6.3 Hardware design and motion control of the SwarmBang robot . . . 8
- 6.4 Division of transmission frequency and customized communication protocol . . . 8

7 Obstacle avoidance strategy in simulation and robotic experiments **8**

8 Estimation of body orientation change over consecutive RGB camera frames in the pybullet simulator **9**

9 Effect of position of the informed individual in the simulation experiments of collective spin and collective turn **10**
- 9.1 Definition of spatial center proximity index . . . 10
- 9.2 Effect on the simulation of collective spin . . . 10
- 9.3 Effect on the simulation of collective turn . . . 11

10 Comparison of robotic experiments between the BOC-based interaction and the Vicsek model 11

11 Impact of group size on BOC-based interaction in simulation experiments of collective spin 11

Supplementary Tables 13

Supplementary Figures 15

Supplementary References 48

Supplementary Notes

1. Data processing of U-turn datasets

In this study, we adopted the U-turn datasets from the groups of rummy-nose tetra (*Hemigrammus rhodostomus*) (1–3) in our empirical analysis, which contains the trajectory with group sizes of 1, 2, 4, 5, 8, 10 fish. For the reliability and generality of the empirical analysis, we chose datasets with the group size as large as possible, i.e., trajectories involving 8 and 10 fish. To remove the outliers and fill in the NaN values in the original datasets, we smoothed the raw trajectory by the Five-point Third-order filtering method.

By using the same U-turn detection method in the Ref. (1), we extracted 44 and 400 U-turn events from the group sizes of 10 and 8, respectively (see Supplementary Movies 1-2 for the replay of the typical U-turn behaviors). As shown in Supplementary Figure 1a and Supplementary Figure 1b, the detection method is based on the sign of group alignment to the wall $\bar{a}(t) = \mathrm{mean}(a_i(t))$, where $a_i(t) = \sin(\theta_{wi}(t))$ denotes the alignment to the wall of fish i. $\theta_{wi}(t) = \phi_i(t) - \theta_i(t)$. $\phi_i(t)$ is the heading of individual i. $\theta_i(t)$ is the angular position of individual i. A collective U-turn begins when the alignment to the wall of the initiating fish i becomes $a_i(t) = 0$. It ends when the alignment to the wall of the last turning fish j becomes $a_j(t) = 0$. After that, we could obtain the exact turning time of each fish and then define the turning order o_i based on the sequential order of turning time moment (Supplementary Figure 1c) in empirical analysis. Particularly, we compiled the trajectory data for each U-turn event into sequences of 350 steps from the original datasets, spanning a total duration of 7 seconds. $\Delta t = 0.02$ in original U-turn datasets. All the trajectories used in this work can be found from Supplementary Figure 3 to Supplementary Figure 8.

2. Analysis of Experimental Data from U-turn behaviors

2.1 Segmentation of U-turn trajectory

The mechanism behind the collective U-turn in groups of rummy-nose tetra (*Hemigrammus rhodostomus*) is highly intricate, involving both informative and misinformative interactions (3). On the one hand, informative interactions are crucial in facilitating directional information transfer in the group. On the other hand, due to limitations in visual perception and potential errors in information transfer, misinformative interactions could also occur in U-turn events. Misinformative interactions lead individuals to respond incorrectly or inconsistently to their neighbors, potentially disrupting the collective U-turn process. These two conflicted interactions may lead to the emergence of a time-varying leader-follower interaction network during the U-turn, which indicates the leadership of each fish might be temporary. Thus, we divided each U-turn trajectory into multiple segments represented by $[T - \tau, T]$ to comprehensively explore the relationship between BOC and leadership. Specifically, we set the minimum time interval of T and τ is 0.2 seconds. Given that the total duration of the U-turn trajectory is $7s$, the time interval of T and τ can be divided into 35 intervals, resulting in a total of 35×35 combinations of $[T - \tau, \tau]$ (see Supplementary Figure 11 for heatmaps composed 35×35 elements).

2.2 Effectiveness of Leadership Evaluation Indicator

To check the correctness of the leadership evaluation indicator defined in the main text Eq. (3), we analyzed the correlation between the leadership L_i and the reciprocal of each individual's turning order $\frac{1}{o_i}$. As shown in Supplementary Figure 2, the results demonstrate the positive correlation between the L_i and the $\frac{1}{o_i}$, implying the evaluation indicator (defined in the main text Eq. (3)) is sufficient to characterize the leadership of each individual during U-turn.

2.3 Spatial Distribution of Turning Order in Fish Schools

In our correlation analysis between the BOC and leadership during the U-turn, we found that the correlation values become strongly positive after involving the individual's frontal preference perception. The reason behind this may be attributed to the fact that the turning information is transmitted from the front-to-back direction. Hence, the consideration of anisotropic perception is necessary in revealing the significant role of BOC during U-turns. We substantiated this evidence by statistically analyzing the spatial distribution of individuals with different turning orders. As shown in Supplementary Figure 9 and Supplementary Figure 10, we found the spatial distribution of the increasing turning order shifts from the front-to-back.

2.4 Distance-based motion salience

To maintain generality in revealing the role of BOC during U-turn, we conducted the same analysis for the well-known motion characteristic, i.e., the distance (4–6). For consistency of mathematical notations in Eq. (1) in the main text, the distance-based motion cue is calculated as follows:

$$d_{ij}(T) = \frac{1}{\|\hat{\mathbf{x}}_i(T) - \hat{\mathbf{x}}_j(T)\|} \cdot \left(\frac{1 + \hat{\mathbf{v}}_i\left(T\right) \cdot \hat{\mathbf{x}}_{ij}\left(T\right)}{2} \right)^{\alpha}, \qquad [1]$$

where $\hat{\mathbf{x}}_i(T)$ and $\hat{\mathbf{x}}_j(T)$ are the position vector of individual i and j at time T, respectively. $\|\cdot\|$ is the vector normalization. The term $\left(\frac{1+\hat{\mathbf{v}}_i(T)\cdot\hat{\mathbf{x}}_{ij}(T)}{2}\right)^{\alpha}$ represents the frontal preference of individual i. α is to adjust the degree of frontal preference. The larger α indicates a greater intention for individuals to select neighbors in the front.

Following the same procedure for deriving the BOC-based motion salience (the detailed procedure is shown in Supplementary Figure 31), we can get the distance-based motion salience. Specifically, we first calculated the relative distance of neighbors from each individual's view at time T according to Eq. (1), which forms a matrix denoted as $\mathbf{D}(T) = [d_{ij}(T)]_{N\times N}$. Then, we derived the distance-based motion salience by averaging each column in $\mathbf{D}(T)$.

2.5 Bearing Change-based motion salience

Plenty of empirical research has revealed that animals select their neighbors based on the bearing change through the visual pathway (7, 8). In this study, we also involved the bearing change as a reference to demonstrate the generality of our empirical findings of the BOC. For consistency of mathematical notations in Eq. (1) in the main text, the bearing change-based motion cue is calculated as follows:

$$b_{ij}(T,\tau) = \frac{\angle\left(\hat{\mathbf{x}}_{ij}\left(T\right), \hat{\mathbf{x}}_{ij}\left(T-\tau\right)\right)}{\tau} \cdot \left(\frac{1 + \hat{\mathbf{v}}_i\left(T\right) \cdot \hat{\mathbf{x}}_{ij}\left(T\right)}{2} \right)^{\alpha}, \qquad [2]$$

where $\hat{\mathbf{x}}_{ij}(T) = (\hat{\mathbf{x}}_j\left(T\right) - \hat{\mathbf{x}}_i(T))/||\hat{\mathbf{x}}_j\left(T\right) - \hat{\mathbf{x}}_i\left(T\right)||$. $\hat{\mathbf{x}}_i\left(T\right)$ and $\hat{\mathbf{x}}_j\left(T\right)$ are the position vector of individual i and j at time T, respectively. τ is the time period. The operator $\angle$ means the angle between two vectors. The term $\left(\frac{1+\hat{\mathbf{v}}_i(T)\cdot\hat{\mathbf{x}}_{ij}(T)}{2}\right)^{\alpha}$ involves the frontal preference into the individual's perception. α is the preference tuning parameter.

Following the same procedure for deriving the BOC-based motion salience (the detailed procedure is shown in Supplementary Figure 31), we can get the bearing change-based motion salience. Specifically, we first calculated the bearing change of neighbors from each individual's view according to Eq. (2), which forms a matrix denoted as $\mathbf{B}(T,\tau) = [b_{ij}(T,\tau)]_{N\times N}$. Then, we averaged each column in $\mathbf{B}(T,\tau)$ to obtain the bearing change-based motion salience.

3. Evaluation Indicators

3.1 Direction of information transfer

Following the definition in the Ref. (9), we defined the direction of information transfer θ_s as follows:

$$\theta_s = \angle(\hat{\mathbf{u}}_s, \hat{\mathbf{v}}_g), \qquad [3]$$

where $\hat{\mathbf{v}}_g = \frac{1}{N}\sum_i^N \hat{\mathbf{v}}_i$ is the group velocity at the moment t_s that the spinning initiator has just spun 2π. $\hat{\mathbf{u}}_s =< \hat{\mathbf{x}}(s_i < 0.2N; t = t_s) > - < \hat{\mathbf{x}}(s_i > 0.8N; t = t_s) >$ is the vector that points from the mean position of individuals who started spinning in the latter 20% to the mean position of individuals who started spinning in the first 20%. s_i is the spinning rank of individual i in the group. $< \cdot >$ represents the average operator. Particularly, $\theta_s = 0°$ suggests that information transfers from the front-to-back, $\theta_s = 90°$ means that it transfers from side-to-side, and $\theta_s = 180°$ indicates the back-to-front transfer direction.

3.2 Polarization

We used the polarization to evaluate the degree of consensus (4), which is defined as follows:

$$\phi = \frac{1}{N}\left\|\sum_{i=1}^{N}\frac{\hat{\mathbf{v}}_i}{\|\hat{\mathbf{v}}_i\|}\right\|, \qquad [4]$$

where N is the group size. $\phi \in [0, 1]$. The higher ϕ indicated the high-level velocity alignment of the group. Conversely, the lower ϕ represents a diffused group, reflecting the lack of alignment in moving direction.

4. Reconstruct the visual field and involvement of visual occlusion from first-person perspective

In this work, we assume that individuals are non-transparent ellipses, meaning that neighbors in close proximity to the focal individual may occlude neighbors that are further away. This occlusion prevents the focal individual from observing the state of distant neighbors within the visual perception range. To do that, we adopted the algorithm provided in the research(10) (codes are kindly provided by the authors in https://zenodo.org/records/4983257)). To reconstruct the visual field and determine the visible and occluded area of neighbors in our simulation, the computation comprised four steps: Firstly, we could identify the positions and angles of the tangent points (black points shown in Supplementary Figure 15a) where the rays originating from the focal individual's eyes (red point shown in Supplementary Figure 15a) touch any neighboring ellipse. Secondly, we calculate the intersection point of rays originating from the eye of the focal individual i through the tangent points on j with the outlines of ellipses k (the red cross shown in Supplementary Figure 15a). If the intersection point exists, it means the occlusion occurs and we then remove the tangent point closest to the intersection point. Otherwise, the absence of intersections implies no occluded individual is in this certain direction. Thirdly, we numerically sort the angular positions of left tangent points for the focal individual in increasing order. Finally, for each segment (i.e., visual field) delimited by two ordered tangent points (light blue regions in Supplementary Figure 15a), we projected the bisectors (purple lines in Supplementary Figure 15a) of these segments to ascertain which ellipse is closest in this segment to find out the belongings. Through computations of these four steps, we are able to distinguish the distant neighbors that are occluded by nearby ones from the first-person perspective of the focal individual.

As shown in Supplementary Figure 15b-c, we compared the impact of the presence or absence of visual occlusion on the process of neighbor selection. Specifically, with the involvement of visual occlusion, distant individuals (colored by the dark grey) are obscured by closer individuals, thus precluding being selected by the focal individual (Supplementary Figure 15b). However, with the absence of the visual occlusion, the focal individual can select any neighbors within its perception range (Supplementary Figure 15c), which is the common perception setup in swarm models, e.g., the metric interaction.

5. Definition of Baseline models

To better demonstrate the advantage of BOC-based interaction, we also involve the Vicsek model as the baseline swarm model in robotic experiments.

5.1 Vicsek Model

In the Vicsek model (4), an individual aligns with the average velocity of its neighbors within the sensing radius R_{visual} at each time step, implying that the focal individual interacts equally with neighbors within its R_{visual}.

The Vicsek model is defined as follows:

$$\theta(t+1) = \langle \theta(t) \rangle_{S_i} + \Delta\theta, \qquad [5]$$

where $\langle \theta(t) \rangle_{S_i}$ denotes the average velocity of neighbors. S_i is the neighbor set of individual i within the the sensing radius R_{visual}. $\Delta\theta$ is the random noise with the uniform distribution. Unless otherwise specified, the parameter selection in the Vicsek model is the same as the model with BOC-based interaction, which can be found in Supplementary Table 3.

6. Details of swarm robotic validation system

To improve the reproducibility of swarm robotic experiments, we first illustrate the architecture and workflow of the swarm robotic validation system. Then, we show the details of how we simulate the visual sensing by the pinhole camera model and estimate the BOC by the simulated images. Additionally, we provide the hardware design and motion control approach of the SwarmBang robot and introduce the customized communication protocol used in the SwarmBang system.

6.1 Architecture and workflow of swarm robotic validation system

As shown in Supplementary Figure 16, we illustrate the architecture of the swarm robotics validation system. The swarm robotics validation system (i.e., SwarmBang system) comprises three primary components: firstly, a server computer; secondly, a motion capture system; and thirdly, a large number of swarm robots (while the system has the capacity to support up to 100 robots, we limited our study to 50 robots due to spatial constraints). The server computer, equipped with a radio transmitter, is tasked with simulating local perception, model computing, and transmitting real-time control commands to the robots. The motion capture system is used to locate the position of each robot. To clarify the differences in computational content between the robots and the server computer, we illustrate the workflow of the swarm robotic validation system in Supplementary Figure 17.

The workflow of our validation system begins with the measurement of each SwarmBang robot's position and heading using the NOKOV motion capture system processed on the server computer (step 1 in Supplementary Figure 17). Next, the server computer simulates the local vision-based

sensing process of each robot through the pinhole camera model (step 2 in Supplementary Figure 17). Based on the pinhole camera model, we can obtain projections of the neighboring robots onto the simulated image plane, allowing us to simulate the visual perception of the robots and approximate the body orientation change (BOC) using the generated bounding boxes based on the simulated images. Then, the server computer determines the desired heading θ_d for each robot based on the corresponding swarm model, utilizing the local visual perception information (step 3 in Supplementary Figure 17). After obtaining the desired heading of each robot, the desired angular speed ω is calculated as the $\omega = \min\left(\frac{\Delta\theta}{\Delta t}, \omega_{\text{max}}^{\text{robot}}\right)$, where the $\Delta\theta = \theta_d(t) - \theta(t)$ and $\omega_{\text{max}}^{\text{robot}}$ is the maximum angular speed in robotic experiment. Following that, the central computer calculates the desired linear and angular velocity of each robot and broadcasts to all robots based on a wireless communication module through the customized communication protocol (see Supplementary Figure 20a for detailed information) with a fixed time interval Δt (step 4 in Supplementary Figure 17). Upon receiving these desired velocities, each robot then calculates the speed of its left and right wheels based on the kinematic model of the differential-drive platform in the robots (step 5 in Supplementary Figure 17).

6.2 Simulate the visual sensing by the pinhole camera model

To further demonstrate the advantage of the BOC-based interaction using local perception information, we simulate the vision-based sensing of the robot by the pinhole camera model. The pinhole camera model is a commonly employed and effective technique for simulating visual sensing process(11–13).

As shown in Supplementary Figure 18, the pinhole model describes the mathematical relationship between the coordinates of a point in three-dimensional space and its projection onto the image plane. Consider a 3D point $p = (p_x, p_y, p_z)$ shown in Supplementary Figure 18a, the position of (u_x, u_y) on the camera plane is calculates as:

$$\begin{aligned} u_x &= f\frac{p_x}{p_z}, \\ u_y &= f\frac{p_y}{p_z}, \end{aligned} \quad [6]$$

where f is the focal length of the camera.

In our robotic experiment, each robot is regarded as an ellipsoid with the same aspect ratio as the real robot. Using the pinhole camera model, we can obtain the projections of robots (ellipsoids) onto the simulated camera's image plane, where the shape of projections varies as the robots' orientation changes (Supplementary Figure 18b-e). To approximate the magnitude of BOC from the simulated images, we used the rectangular formed by the maximum X and Y ranges of the robot's projection as the bounding box (red rectangle in Supplementary Figure 18b-e) and estimated the BOC by the variation in the area of the bounding box. The relationship between the robot's orientation and the corresponding area of the simulated bounding box is depicted in Supplementary Figure 18f. From Supplementary Figure 18f, we observed that as the robot's orientation continuously changes, the area of the bounding box on the simulated image plane also increases steadily, which provides evidence that the variation in the area of the simulated bounding box is practical to estimate the BOC of neighboring robots. To streamline the robotic experiments, we assume the robot has omnidirectional vision, which is a common hardware setup in vision-based swarm robotics(14–17).

6.3 Hardware design and motion control of the SwarmBang robot

The schematic of the robot's hardware architecture is shown in Supplementary Figure 19. The hardware architecture of the SwarmBang robot is separated into two key parts: 1) the PCB board for decision-making and communications and 2) the PCB board for motion control and battery management. Two 3.7V rechargeable batteries (2∗800mAh) provide energy for about 1 hour in our experimental settings. Each robot is equipped with a wireless communication module (NRF24L01) to receive commands from the server computer. According to the wheeled robot's kinematics, after receiving the motion command of the desired linear and angular speed, the velocities of the left and right wheels are calculated as follows:

$$\begin{aligned} v_l &= v + \frac{\omega}{2} \cdot L, \\ v_r &= v - \frac{\omega}{2} \cdot L, \end{aligned} \qquad [7]$$

where the v and ω are the linear speed and angular speed, respectively. L is the distance between the left and right wheels of robots.

To control the velocity of the robot's wheels, we use four-phase, five-wire step motors to drive the SwarmBang robots. The inherent step angle of the motor shaft is 5.625 degrees, with a subdivision ratio of 64. It takes 4096 pulse signals for the motor shaft to rotate the robot's wheel one full revolution. The rotational speed of the step motor is adjusted by changing the frequency of the pulse signals. The maximum pulse frequency that the step motor can respond to is 1 kHz, resulting in a maximum motor speed of approximately 14.65 RPM ($v_{\mathrm{rpm}}^{\max} = \frac{f\times 60}{\frac{360}{5.625}\times 64} = \frac{1000\times 60}{4096} \approx 14.6\,\mathrm{RPM} \approx 0.24\,\mathrm{n\,s^{-1}}$). The wheels of the SwarmBang robots have a radius of $r = 17.5$mm, and the distance between the left and right wheels is $L = 60$mm. Therefore, the theoretical maximum linear speed of the real robot is approximately $v \approx 25\ \mathrm{mm\,s^{-1}}$, and the theoretical maximum angular velocity is approximately $\omega_{\max}^{\mathrm{robot}} \approx 0.83\,\mathrm{rad\,s^{-1}}$.

6.4 Division of transmission frequency and customized communication protocol

Transmitting motion commands for 50 robots with low communication bandwidth and tight latency requirements poses significant challenges for the robotic validation system. Hence, we developed a low-redundancy communication protocol and employed multiple communication frequencies to simultaneously transmit motion commands, with each frequency corresponding to 25 robots. As shown in Supplementary Figure 20a, each robot's desired linear and angular speeds are represented using 9 bits: 1 bit for indicating the turning direction (0 for left, 1 for right), 3 bits for the desired linear speed, and 5 bits for the angular speed. Each data frame contains the desired linear and angular speeds for 25 robots, totaling $9 \times 25 = 225$ bits. Additionally, we have implemented a Cyclic Redundancy Check (CRC) in each robot to verify the correctness of the received data. Upon receiving a data frame, each robot locates its desired linear and angular speeds within the data frame based on its unique ID. In particular, we employed frequency division multiplexing (2.42GHz and 2.46GHz) to separately control two groups of robots based on the customized communication protocol, each consisting of 25 robots (Supplementary Figure 20b), totaling 50 robots. The method of frequency division enhances communication bandwidth and enables the control of large-scale swarm robotics to validate the feasibility of the BOC-based interaction.

7. Obstacle avoidance strategy in simulation and robotic experiments

For both the simulation of collective turn and robot experiments, we involved the commonly used soft-repulsion term into the interaction rule (defined in main text Eq. (7)) to avoid collisions among

individuals. The soft-repulsion term is defined as follows:

$$\hat{\mathbf{v}}_{\text{rep},i}^{\text{soft}} = \begin{cases} \dfrac{(d_{\text{soft}}^{\text{ind}} - d_{ij})}{d_{ij}} (\hat{\mathbf{x}}_i - \hat{\mathbf{x}}_j) & \text{if } d_{ij} < d_{\text{soft}}^{\text{ind}} \\ \mathbf{0} & \text{otherwise} \end{cases}, \qquad [8]$$

where $\hat{\mathbf{x}}_i$ and $\hat{\mathbf{x}}_j$ are the position vector of individual i and j, respectively. d_{ij} is the distance between the individual i and j. $d_{\text{soft}}^{\text{ind}}$ is the threshold distance that triggers an individual to move away from others.

Due to the robot's motion error and the control latency, we also involved the hard-repulsion term to minimize collisions among robots. Both hard and soft repulsion share the same mathematical formulation. The difference lies in the distance threshold that activates the repulsion between individuals. Specifically, the hard-repulsion is engaged at a shorter distance among individuals exclusively when the neighboring robots are in front of the focal one. Besides that, upon activation of the hard-repulsion, the individual also reduces its speed to v_0^{min}. The hard-repulsion term is defined as follows:

$$\hat{\mathbf{v}}_{\text{rep},i}^{\text{hard}} = \begin{cases} \dfrac{(d_{\text{hard}}^{\text{ind}} - d_{ij})}{d_{ij}} (\hat{\mathbf{x}}_i - \hat{\mathbf{x}}_j) & \text{if } d_{ij} < d_{\text{hard}}^{\text{ind}} \text{and} \left(\frac{1+\hat{\mathbf{v}}_i(t)\cdot\hat{\mathbf{x}}_{ij}(t)}{2}\right) \in [0.5, 1] \\ \mathbf{0} & \text{otherwise} \end{cases}, \qquad [9]$$

where $d_{\text{hard}}^{\text{ind}}$ is the distance threshold in the hard-repulsion term. $\left(\frac{1+\hat{\mathbf{v}}_i(t)\cdot\hat{\mathbf{x}}_{ij}(t)}{2}\right)$ represents the relative bearing of neighbor j. When the value is greater than 0.5 and less than 1, it indicates that the neighbor j is in front of the focal individual in the range of $[-\pi/2, \pi/2]$.

According to the above definitions of repulsion terms, we prioritized the hard-repulsion as the primary interaction in our swarm model. Hence, the velocity of the individual i in the collective turn is updated as follows:

$$\hat{\mathbf{v}}_i(t+1) = (1-\mu(t))\left(\hat{\mathbf{v}}_i(t) + k_a \cdot \sum_{j\in S_i} \mathbf{\Theta}\left(g_{ij} = \max(\mathbf{M}_i)\right)\cdot \hat{\mathbf{v}}_j(t) + k_{\text{soft}}^{\text{ind}} \cdot \hat{\mathbf{v}}_{\text{rep},i}^{\text{soft}}\right) + \mu(t)\cdot k_{\text{hard}}^{\text{ind}} \cdot \hat{\mathbf{v}}_{\text{rep},i}^{\text{hard}}, \qquad [10]$$

where $\mu(t) = 1$ indicates that the hard-repulsion is activated at time t, otherwise $\mu(t) = 0$. k_a is the gain of velocity alignment. $k_{\text{soft}}^{\text{ind}}$ is the gain of soft-repulsion term. $k_{\text{hard}}^{\text{ind}}$ is the gain of hard-repulsion term. S_i represents the neighbor set of the focal individual i. $\mathbf{M}_i = [g_{i1}(T,\tau), ..., g_{ij}(T,\tau)]$ comprises the magnitude of BOC observed from the individual i. The magnitude of BOC $g_{ij}(T,\tau)$ is defined in the main text Eq. (4). Heaviside function $\mathbf{\Theta}$ takes a value of 1 when its argument $g_{ij} = \max(\mathbf{M}_i)$. Otherwise, $\mathbf{\Theta}$ takes a value of 0.

8. Estimation of body orientation change over consecutive RGB camera frames in the pybullet simulator

To validate the feasibility of estimating the BOC on robots using visual perception, we intend to estimate the BOC by bounding boxes of neighbors over consecutive frames in the pybullet simulator. First, the pybullet simulator has both a built-in OpenGL GPU visualizer and a built-in CPU renderer, which makes it easy for us to obtain the simulated RGB images from the first-person perspective of robots. By recognizing the color and shape of the robot, we could obtain the bounding

box of each robot from simulated images in the pybullet. The bounding box (x, y, w, h) is defined as a rectangular characterized by four parameters: the x and y coordinates of the top-left corner in the image, the box's width (w), and the box's height (h), which represents the position and size of an object within an image.

Second, we conducted the simulated robotic experiments with a group size of 10 to get the consecutive simulated images from a certain robot's view (Supplementary Figure 21a), where the informed individual is the robot-0 and initiate the abrupt turning at the 50s. As shown in Supplementary Figure 21b, we demonstrated the simulated camera view of robot-2 from the $T = 51s$ to $T = 54s$ and marked the bounding boxes (red boxes in Supplementary Figure 21b) of four neighboring robots detected from the simulated RGB images. Third, to quantify the approximated BOC based on these bounding boxes, it is intuitive to estimate BOC by measuring the changes in the bounding box area. On the one hand, the bounding box area is related to the body orientation change of individuals. On the other hand, estimating BOC through bounding box areas is computationally simpler and more efficient.

Finally, as shown in Supplementary Figure 21c, we estimate the BOC based on the area of the bounding boxes over consecutive simulated RGB images. From the Supplementary Figure 21c, we found that before the informed individual made the abrupt turn, the estimated BOC remained low and stable for all the perceived robots (robots 0, 8, 6, and 9). After the informed individual suddenly turned, the BOC of robot-0 (the informed individual) is the first to increase, followed by a rising trend in the BOC of the other three robots. These results suggested that changes in the bounding box area can be used to quantitatively reflect the trend of BOC.

9. Effect of position of the informed individual in the simulation experiments of collective spin and collective turn

9.1 Definition of spatial center proximity index

To investigate the impact of the informed individual's (or spin/turn initiator) position on the simulation experiments of collective spin and collective turn, we first defined the spatial center proximity index γ to characterize the relative position of informed individual respect to the flock center.

As shown in Supplementary Figure 22a, the γ is calculated as the $\gamma = \frac{d^c}{\mathrm{F_{radius}}}$, which is the ratio of the relative distance between the initiator and the center to the radius of the flock. d^c is the relative distance between the initiator and the center. $\mathrm{F_{radius}}$ is the radius of the flock, estimated by half of the maximal relative distance among individuals. When the $\gamma \leq 0.45$, the initiator is positioned around the flock center (Supplementary Figure 22b). When the $0.45 < \gamma < 0.75$, the initiator is positioned around the middle of the flock (Supplementary Figure 22c). When the $\gamma \geq 0.75$, the initiator is positioned around the border of the flock (Supplementary Figure 22d).

9.2 Effect on the simulation of collective spin

We demonstrate the simulation results of collective spin with different positions of informed individuals in Supplementary Figure 23. We analyzed the impact of the informed individual's position on information propagation from four perspectives: the emergence of scale-free correlation, changes in information transfer speed, the maximum spinning lag, and group polarization. As shown in Supplementary Figure 23a-c, we found that the position of initiators has negligible impact on the simulation outcome of collective spin. Specifically, the correlation length linearly increases with the flock size when the initiator is positioned around the center, middle and border of the group,

indicating the emergence of scale-free correlation regardless of the informed individual's position. In addition, with the increasing group size, the information transfer speed and the max spinning lag also grow and the group polarization shows the decreasing tendency, which is consistent with the results demonstrated in the main text Fig.3.

9.3 Effect on the simulation of collective turn

Simulation results of collective turn with different positions of informed individuals are shown in Supplementary Figure 24. We compare the results from the response accuracy, responsiveness, change in information transfer speed, and the emergence of scale-free correlation. We found that the simulation outcome of collective turn is barely affected by the initiator's position. No matter whether the initiator is located at the center, middle, or border of the group, the group not only exhibits a high response accuracy in quickly responding to the initiator's sudden turns (Supplementary Figure 24a-b), but also demonstrates the emergence of scale-free correlation within the group (Supplementary Figure 24c-e). Additionally, the information transfer speed shows an increasing trend with the increase in group size (Supplementary Figure 24d-f). These results are consistent with those presented in the main text Fig.4.

10. Comparison of robotic experiments between the BOC-based interaction and the Vicsek model

The experimental results demonstrate that using the BOC-based interaction, the swarm with 50 robots not only successfully follows the informed robot to change its trajectory but also quickly responds to the heading change of the informed robot (Supplementary Figure 25a). As a comparison, the swarm using the Vicsek model is difficult to follow the informed robot (Supplementary Figure 25b). As shown in Supplementary Figure 25c and Supplementary Figure 25d, there are noticeable differences in accuracy and responsiveness between BOC-based interaction and the Vicsek model. The response accuracy as a function of time is shown in Supplementary Figure 25e. Furthermore, as shown in Supplementary Figure 25f, the V_s of BOC is much faster than that of the Vicsek model, which is the reason behind the high responsiveness and accuracy of BOC-based interaction (Supplementary Figure 25f). These experiment results highlight the advantage of BOC-based interaction in facilitating the emergence of collective response in swarm robotics.

11. Impact of group size on BOC-based interaction in simulation experiments of collective spin

To demonstrate the basic property of information transfer in the simulation experiments of collective spin, we investigated the impact of group size on BOC-based interaction from the view of group polarization, max spinning lag, and direction of information transfer. As the group size increased, we found a decreasing trend in the group's polarization (Supplementary Figure 30a) and higher maximal spinning lag (Supplementary Figure 30b) which is defined as the time that the last individual starts to spin lag behind the initiator (see Supplementary Note 3 for the detailed definition). Besides that, we also analyzed the direction of information transfer θ_s within the group, which provides valuable insights into how information travels within the group. θ_s is approximated by the angle between the group velocity and the vector that points from the mean position of individuals who started spinning in the latter 20% to the mean position of individuals who started spinning in the first 20% (see Supplementary Note 3 for the detailed definition). Particularly, $\theta_s = 0°$ suggests that information transfers from the front-to-back, $\theta_s = 90°$ means that it transfers from side-to-side, and $\theta_s = 180°$

indicates the back-to-front transfer direction. As shown in Supplementary Figure 30c, as the group size increases, the decreasing trend of θ_s implies that the flow of information transfer becomes more aligned with the front-to-back direction of the simulation setup.

Supplementary Tables

Supplementary Table 1 | Model parameters selection in simulation experiments of collective spin

parameter	value	unit	parameter	value	unit
max simulation step	60	step	Δt	0.2	second
v_0^{min}	0	$\mathrm{mm\,s^{-1}}$	v_0	0	$\mathrm{mm\,s^{-1}}$
R_{visual}	600	mm	$\omega_{\text{max}}^{\text{sim}}$	0.83	$\mathrm{rad\,s^{-1}}$
τ	5	step	activate time	25	step

Supplementary Table 2 | Model parameters selection in simulation experiments of collective turn

parameter	value	unit	parameter	value	unit
k_a	2	null	max simulation step	200	step
$k_{\text{soft}}^{\text{ind}}$	1	null	θ_{info}	$\frac{\pi}{2}$ or π	rad
$k_{\text{hard}}^{\text{ind}}$	1	null	v_0	15	$\mathrm{mm\,s^{-1}}$
$d_{\text{soft}}^{\text{ind}}$	150	mm	v_0^{min}	2	$\mathrm{mm\,s^{-1}}$
$d_{\text{hard}}^{\text{ind}}$	90	mm	$\omega_{\text{max}}^{\text{sim}}$	0.33	$\mathrm{rad\,s^{-1}}$
R_{visual}	600	mm	τ	10	step
Δt	1	second	activate time	50	step

Supplementary Table 3 | Model parameters selection in swarm robotic experiments

parameter	value	unit	parameter	value	unit
k_a	2	null	R_{visual}	750	mm
$k_{\text{soft}}^{\text{ind}}$	1	null	τ	5	step
$k_{\text{hard}}^{\text{ind}}$	1	null	v_0	15	$\mathrm{mm\,s^{-1}}$
$d_{\text{soft}}^{\text{ind}}$	375	mm	v_0^{min}	2	$\mathrm{mm\,s^{-1}}$
$d_{\text{hard}}^{\text{ind}}$	225	mm	$\omega_{\text{max}}^{\text{robot}}$	0.67	$\mathrm{rad\,s^{-1}}$
Δt	0.5	second	activate time	100	step

Supplementary Figures

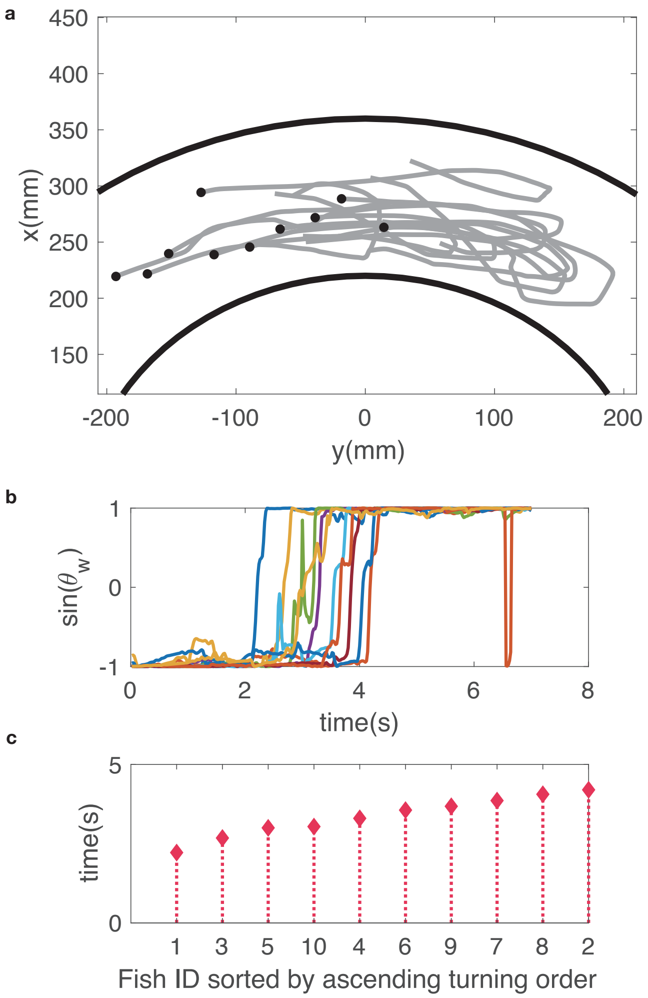

Supplementary Figure 1 | The U-turn trajectory (a) is the same as the Fig.2a in the main text. (b) Normalized degree of alignment with the wall $a_i(t) = \sin(\theta_{wi}(t))$ as a function of time. (c) The turning time of each individual during the U-turn is sorted by the ascending turning order.

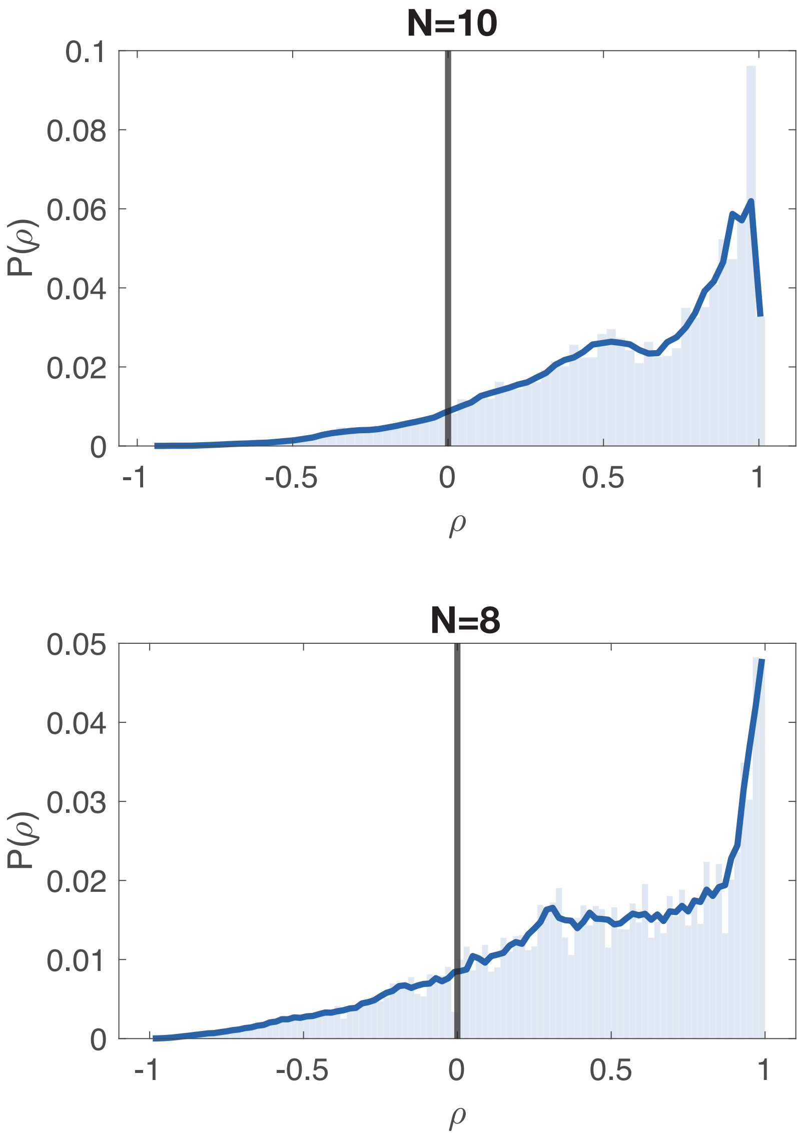

Supplementary Figure 2 | Correlation analysis between the leadership L_i and the reciprocal of turning order $\frac{1}{o_i}$ for $N = 8$ and $N = 10$, respectively.

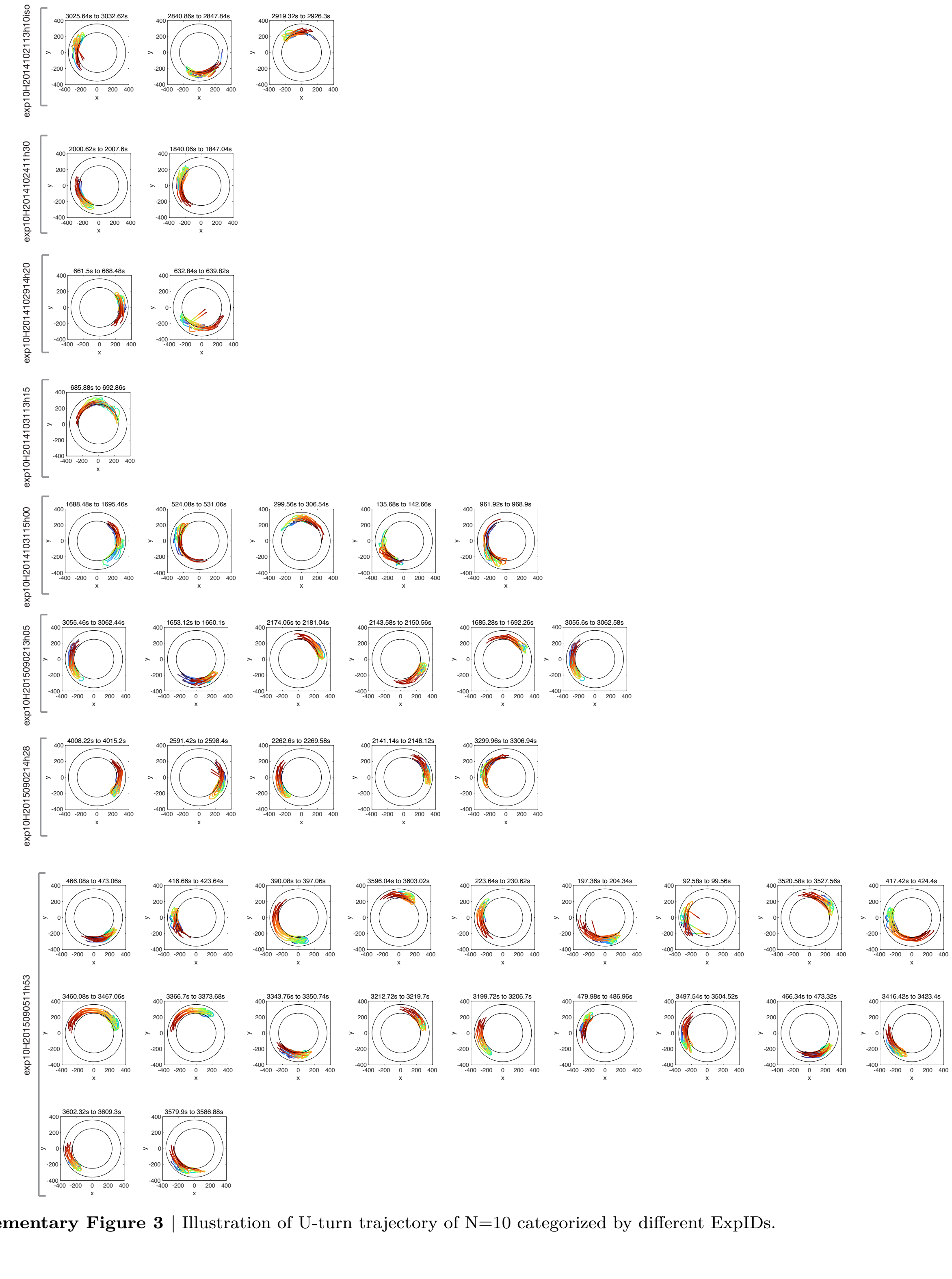

Supplementary Figure 3 | Illustration of U-turn trajectory of N=10 categorized by different ExpIDs.

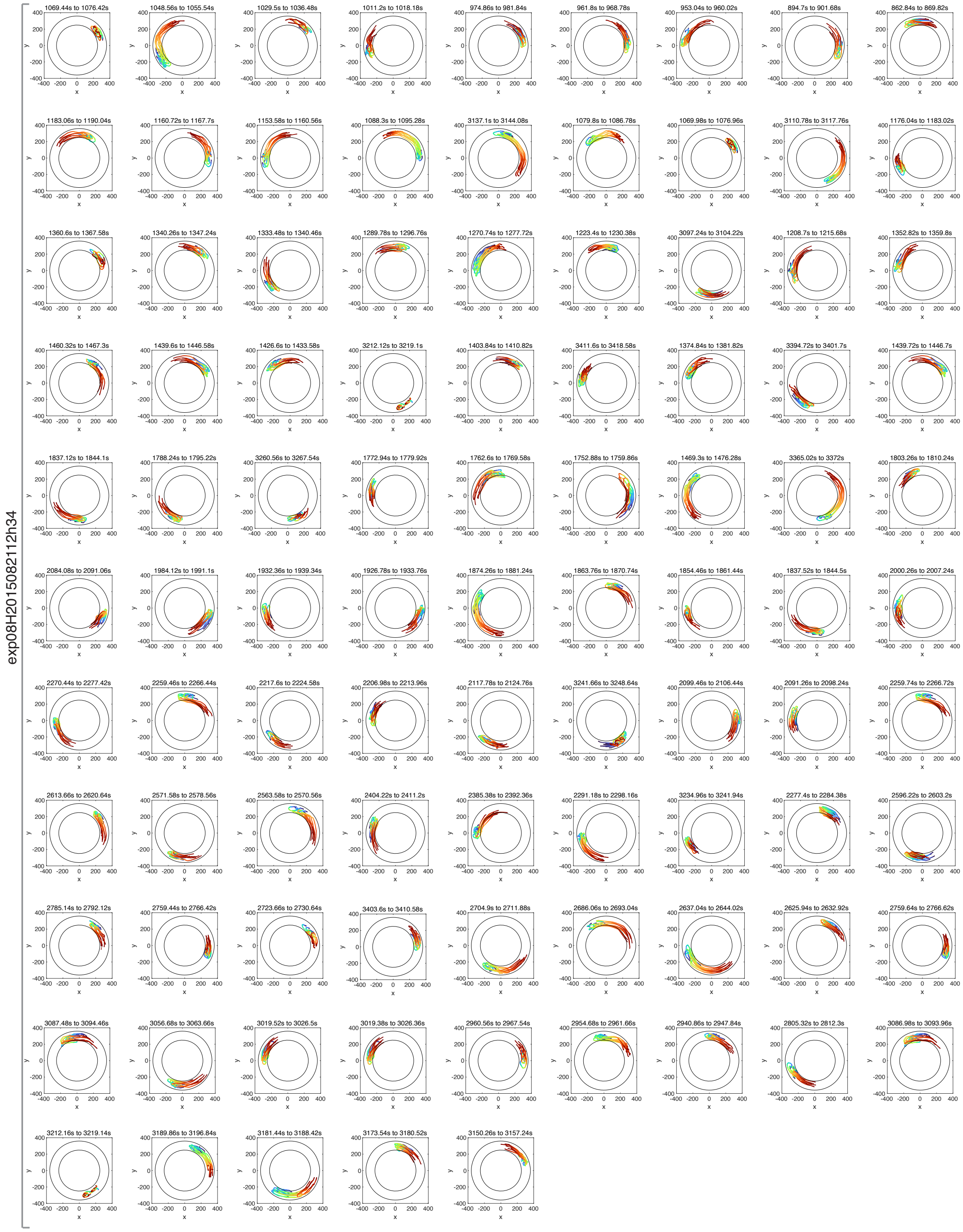

Supplementary Figure 4 | Illustration of U-turn trajectory of N=8 categorized by different ExpIDs.

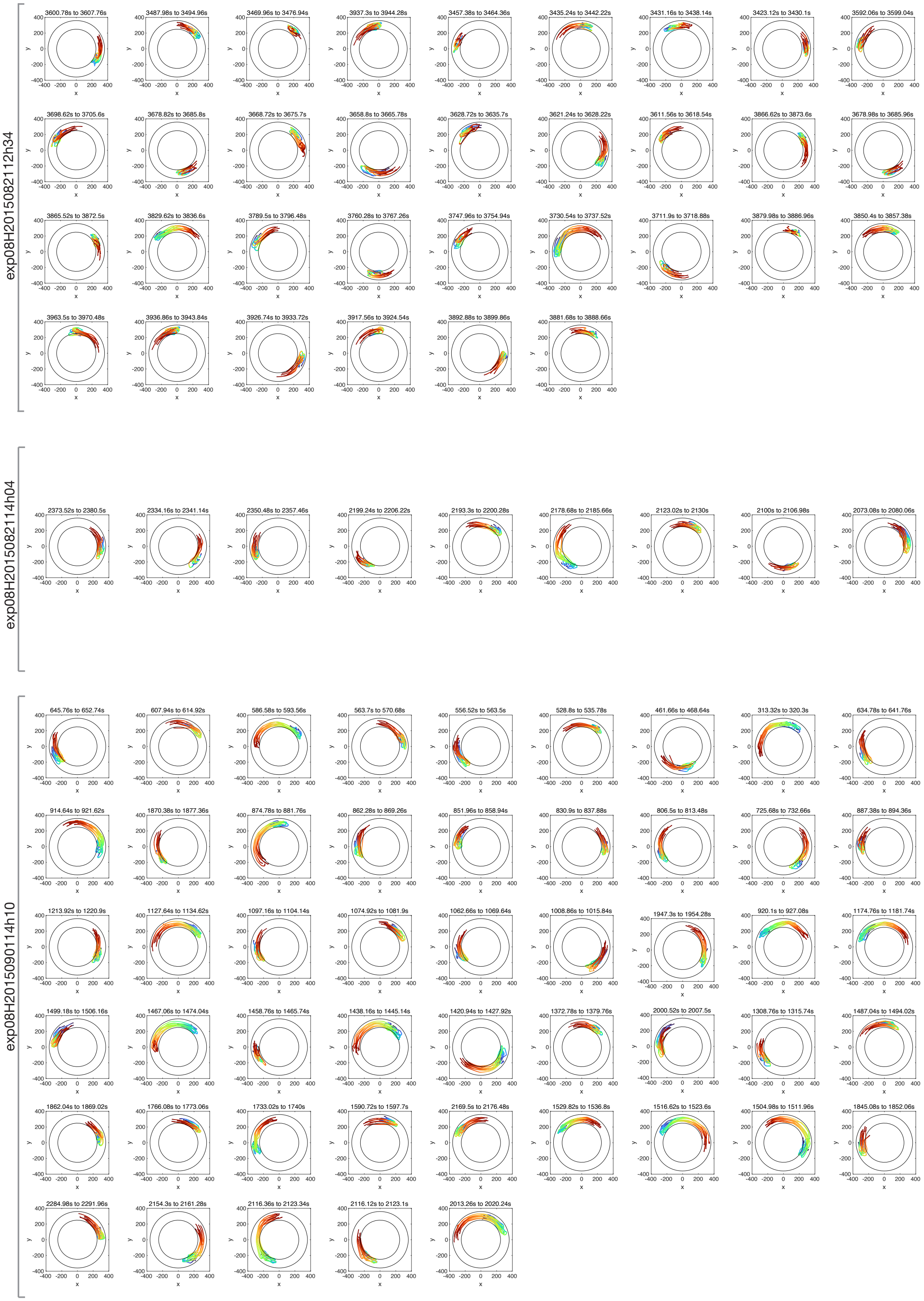

Supplementary Figure 5 | Illustration of U-turn trajectory of N=8 categorized by different ExpIDs.

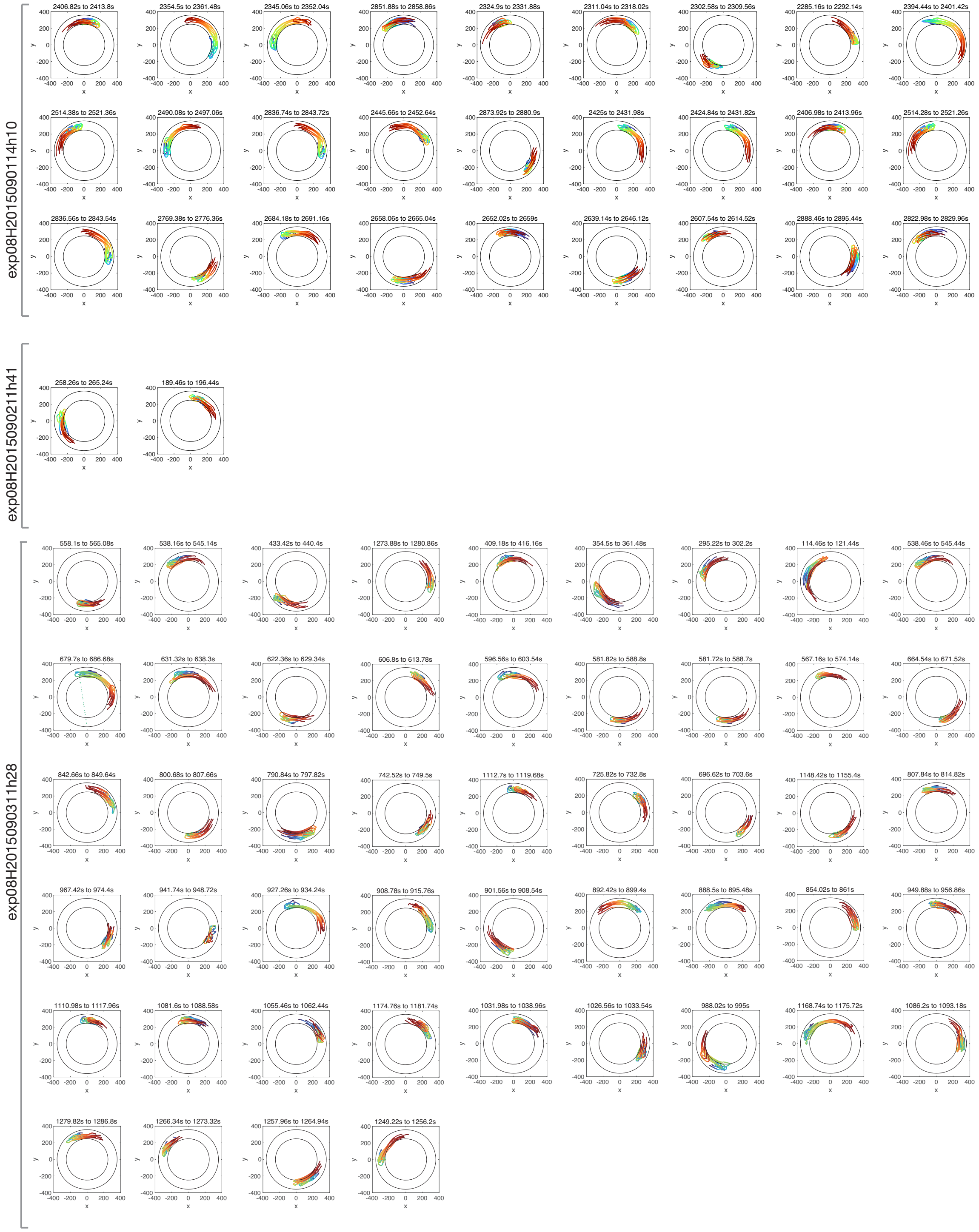

Supplementary Figure 6 | Illustration of U-turn trajectory of N=8 categorized by different ExpIDs.

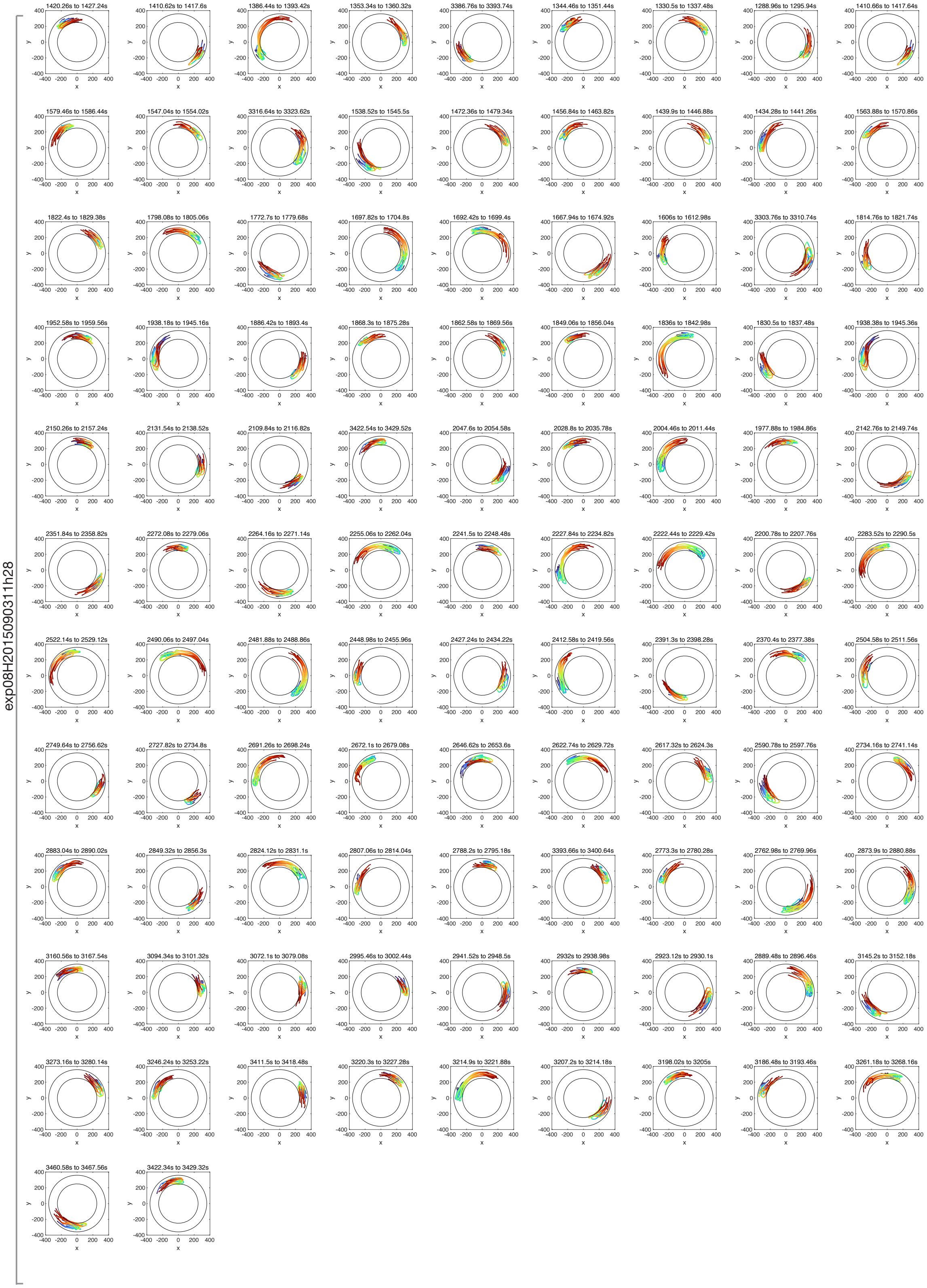

Supplementary Figure 7 | Illustration of U-turn trajectory of N=8 categorized by different ExpIDs.

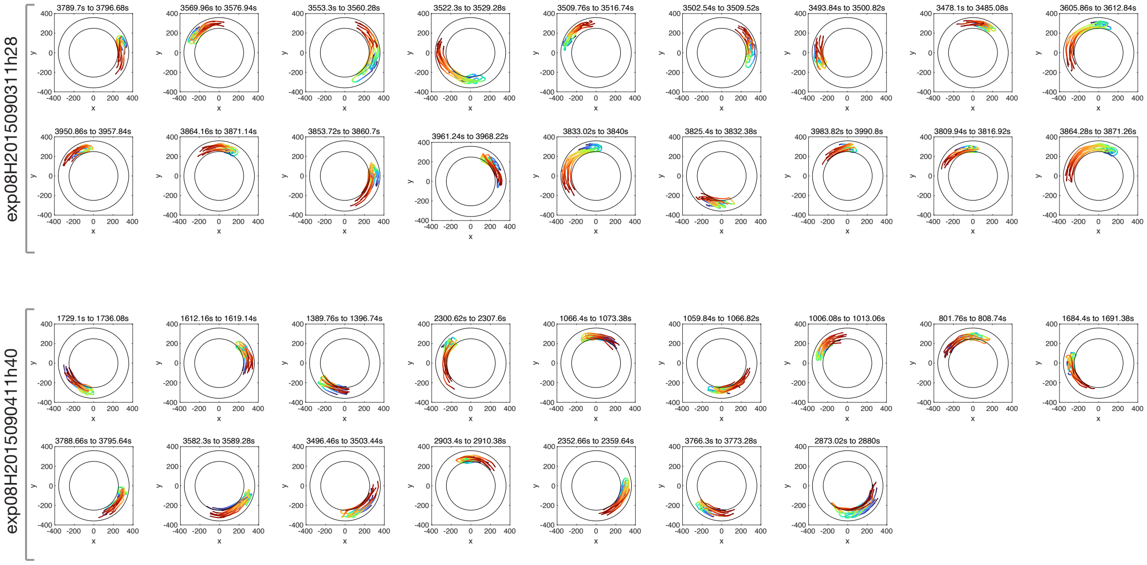

Supplementary Figure 8 | Illustration of U-turn trajectory of N=8 categorized by different ExpIDs.

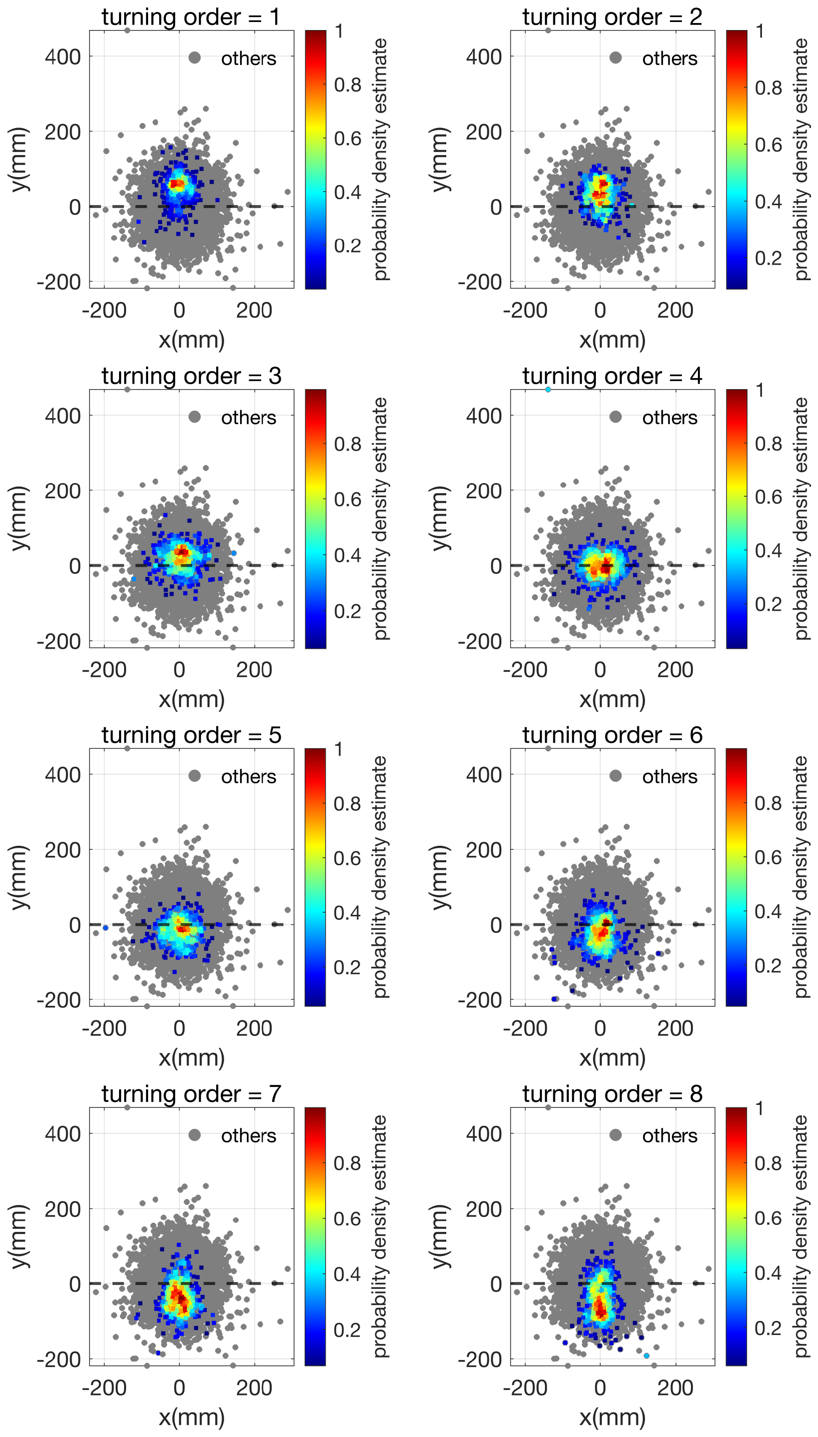

Supplementary Figure 9 | Spatial distribution of different turning orders in the group with $N = 8$ fish.

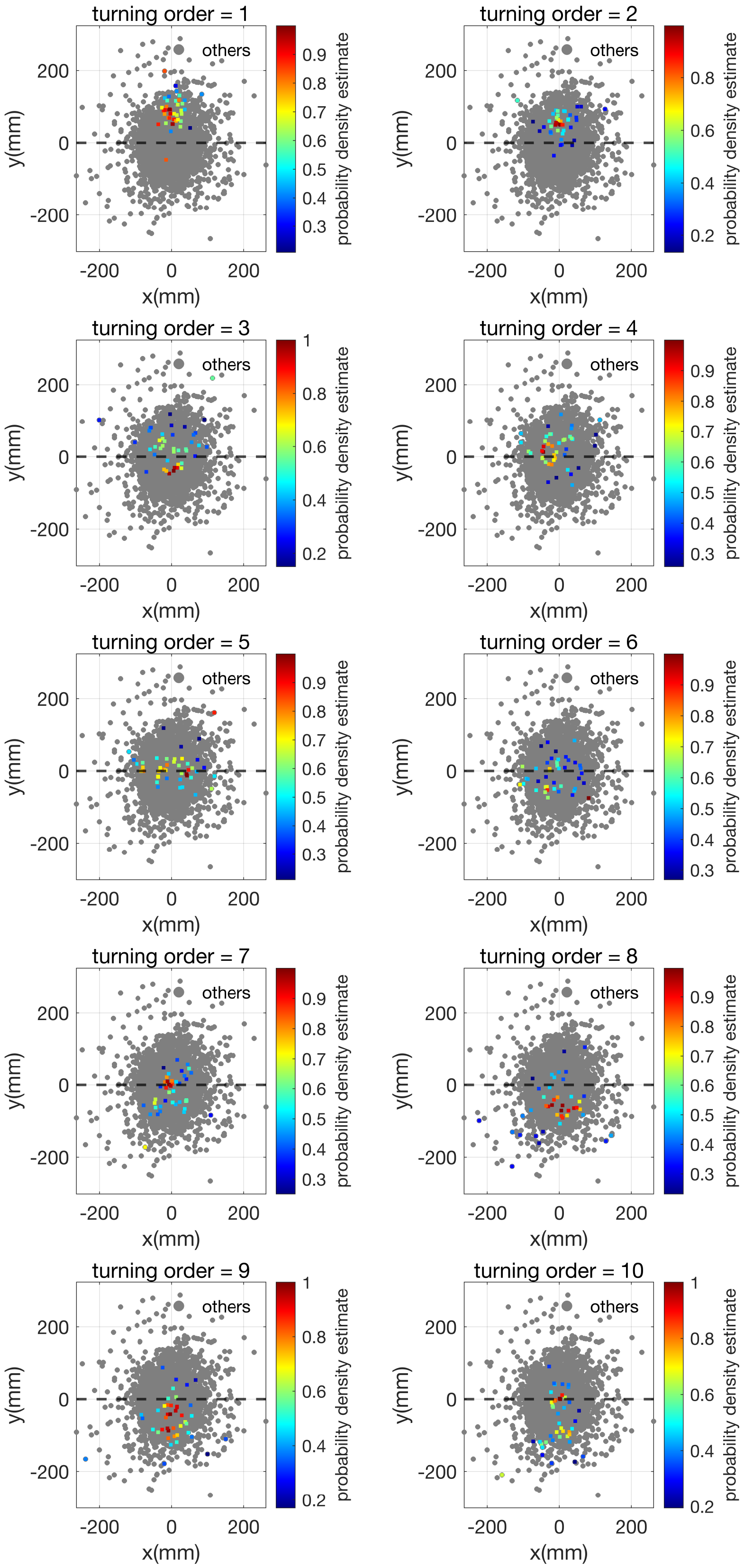

Supplementary Figure 10 | Spatial distribution of different turning orders in the group with $N = 10$ fish.

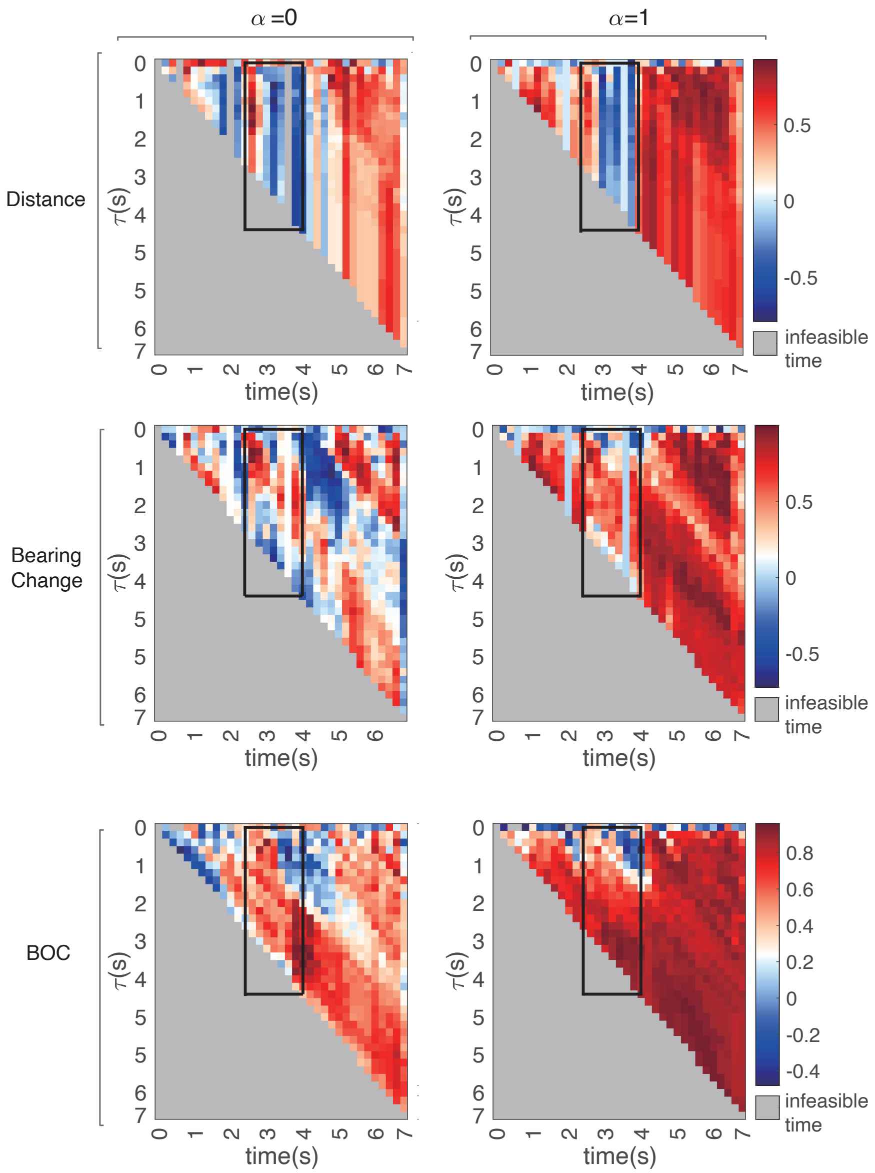

Supplementary Figure 11 | Heatmaps of ρ with $\alpha = 0$ and $\alpha = 1$ for the distance, bearing change and BOC-based interaction.

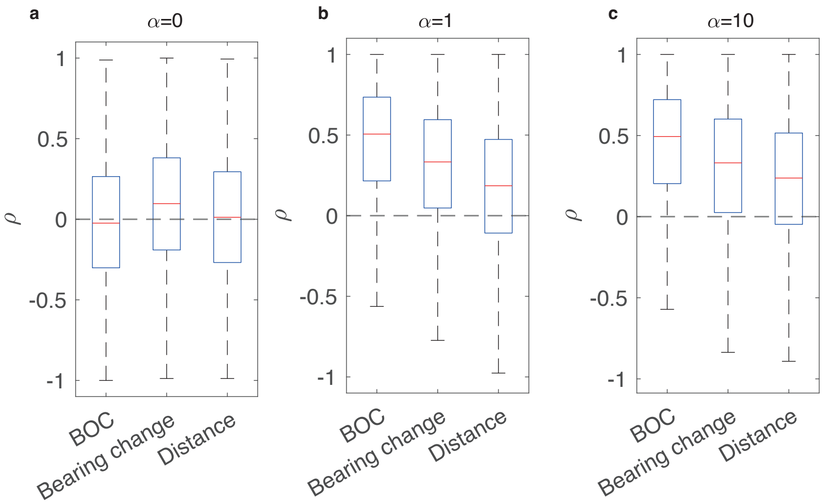

Supplementary Figure 12 | Boxplot of ρ with BOC, bearing change and distance, respectively. The red line in each boxplot is the mean value of the ρ.

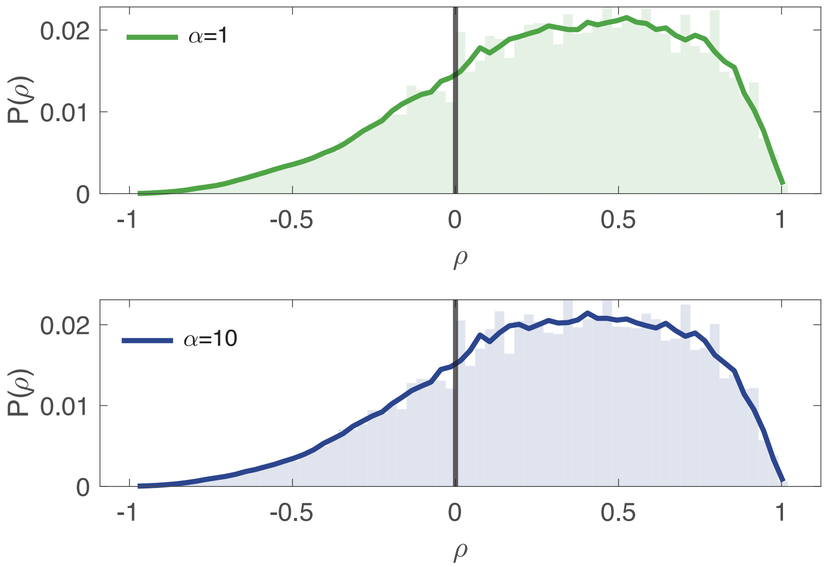

Supplementary Figure 13 | Correlation analysis between the leadership and the front preference only. The peak of ρ is only around 0.3-0.5, which is less than the peak of the combination with the BOC.

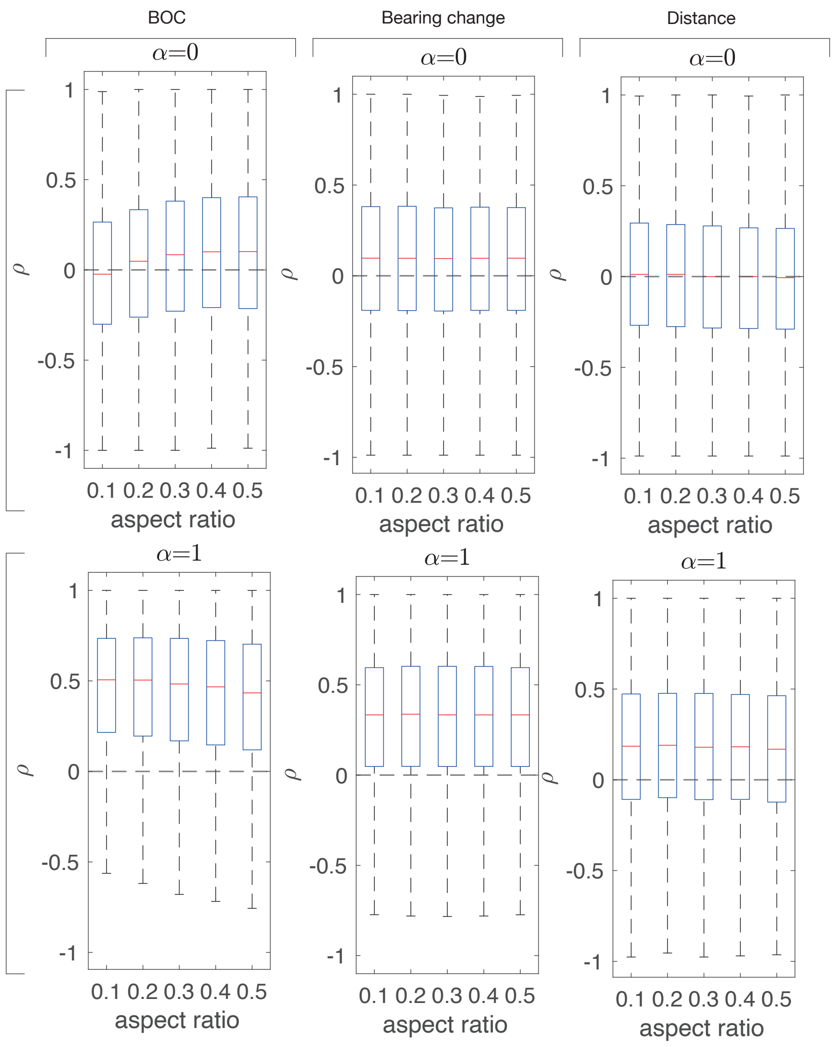

Supplementary Figure 14 | The effect of aspect ratio on the Spearman correlation analysis of BOC, bearing change, and distance-based interaction with the leadership, respectively. It is evident that the variation of the individual's aspect ratio has little impact on the empirical analysis results. The red line in each boxplot is the mean value of the ρ.

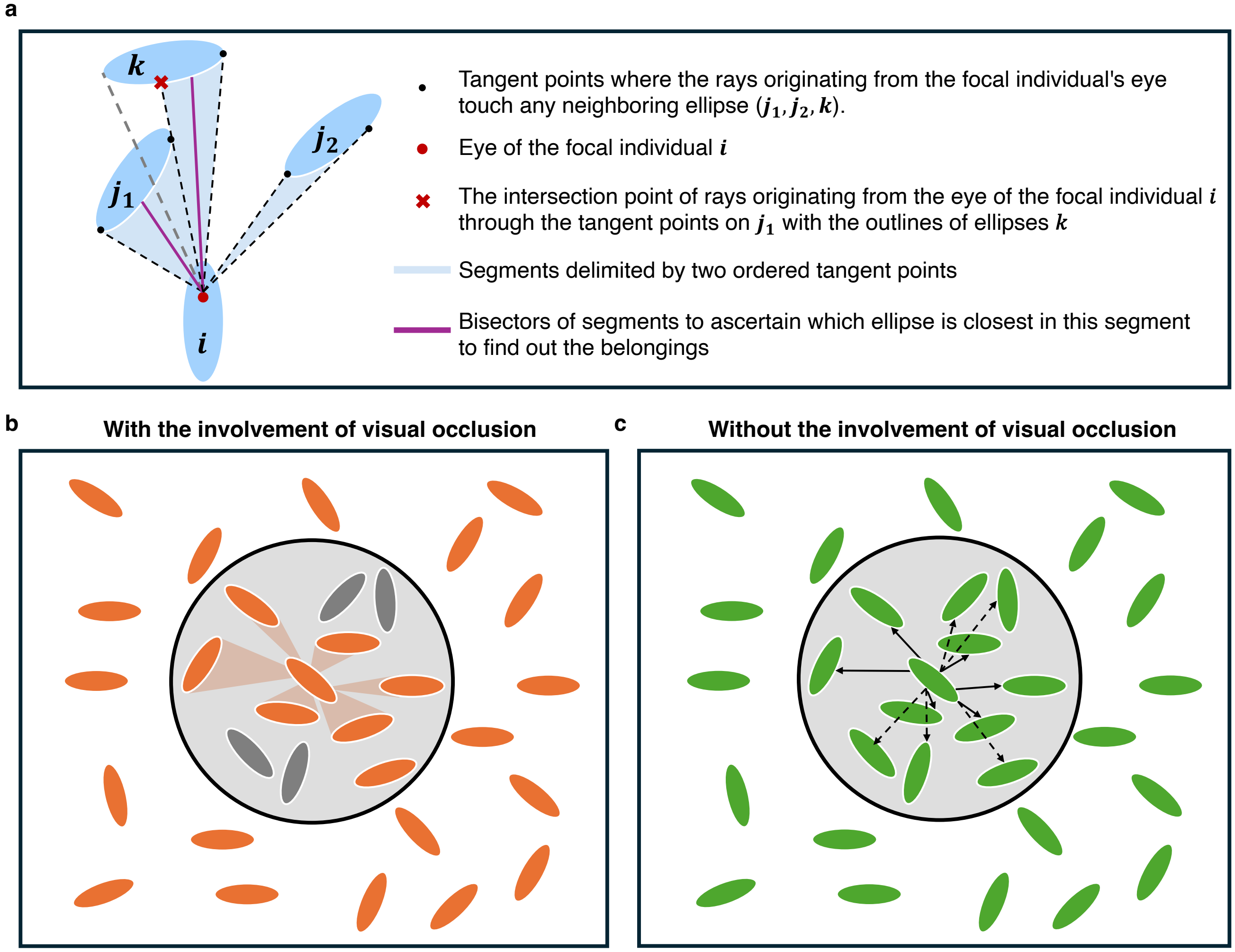

Supplementary Figure 15 | The reconstruction of visual field and involvement of visual occlusion. In this work, we assume that individuals are non-transparent ellipses, implying that neighbors in close proximity to the focal individual may occlude neighbors that are further away. This occlusion prevents the focal individual from perceiving the distant neighbors within the visual perception range. a, we demonstrate how we determine the occluded neighbors from the first-person perspective of individuals. Firstly, we could identify the positions and angles of the tangent points (black points shown in panel a) where the rays originating from the focal individual's eyes (red point shown in a) touch any neighboring ellipse. Secondly, we calculate the intersection point of rays originating from the eye of the focal individual i through the tangent points on j with the outlines of ellipses k (the red cross shown in panel a). If the intersection point exists, it means the occlusion occurs and we then remove the tangent point closest to the intersection point. Otherwise, the absence of intersections implies no occluded individual is in this certain direction. Thirdly, we numerically sort the angular positions of left tangent points for the focal individual in increasing order. Finally, for each segment (i.e., visual field) delimited by two ordered tangent points (light blue regions in panel a), we projected the bisectors (purple lines in panel a) of these segments to ascertain which ellipse is closest in this segment to find out the belongings. Through computations of these four steps, we are able to distinguish the distant neighbors that are occluded by nearby ones from the first-person perspective of the focal individual. The computational process in panel a is proposed in Ref.(10). b-c, For groups with the same spatial distributions, we compared the impact of the presence or absence of visual occlusion on the process of neighbor selection. Specifically, with the involvement of visual occlusion, distant individuals (colored by the dark grey) are obscured by closer individuals, thus precluding being selected by the focal individual (panel b). However, with the absence of the visual occlusion, the focal individual can select any neighbors within its perception range (panel c), which is the common perception setup in swarm models, e.g., the metric interaction.

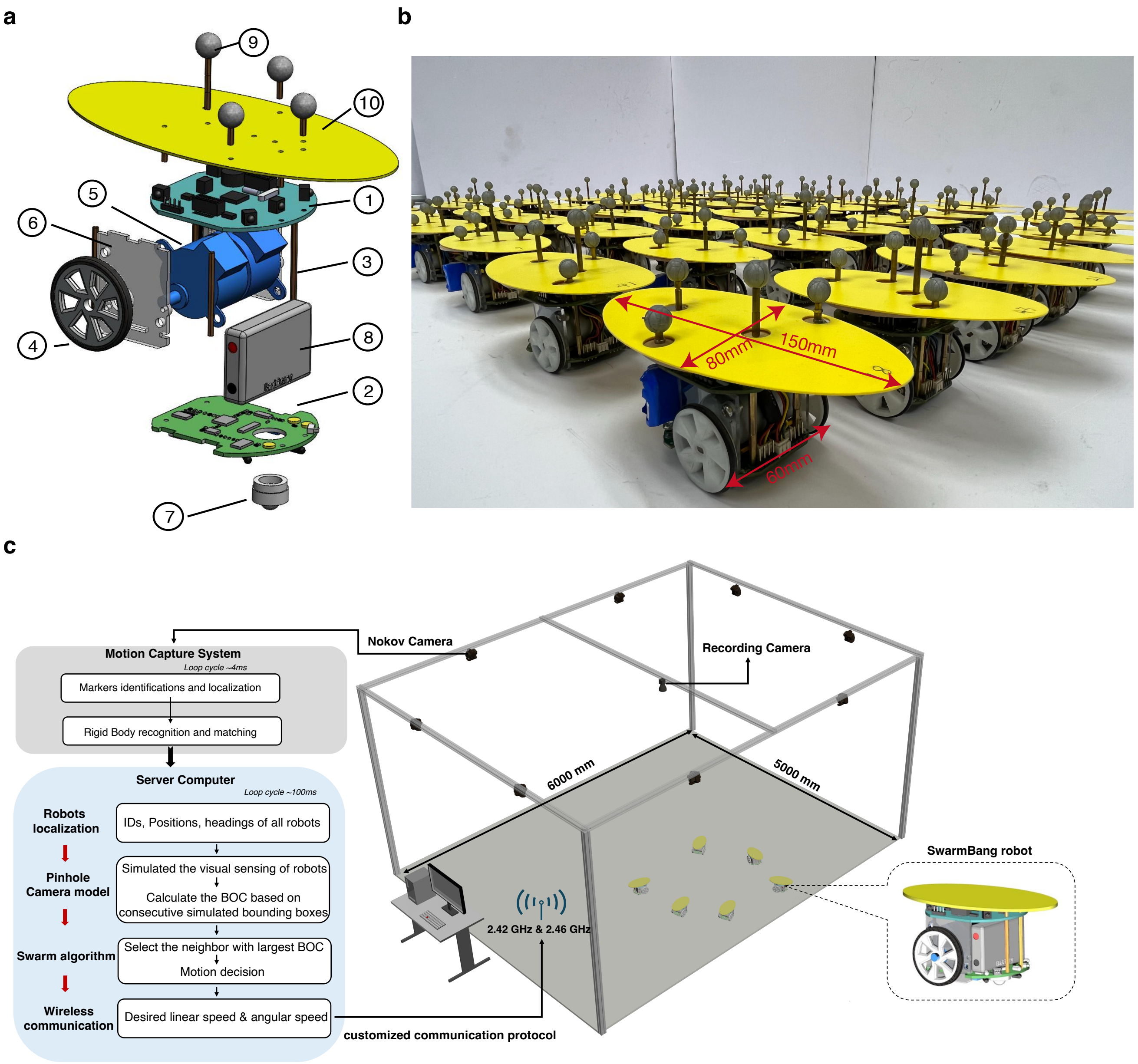

Supplementary Figure 16 | Experimental set-up of the swarm robotics system. a, Overview of the miniature mobile robot (i.e., SwarmBang robot) used for swarm experiments. The robot is a miniature platform (60mm × 60mm × 60mm) and weighs 200g. The components of a robot are described as follows (numbers between parentheses refer to labels in (a)). The robot is built with two-layered structures to keep in line with the sensor-reaction loop in the swarm model, i.e., top sensor PCB board (1) and bottom actuation PCB board (2). They are directly connected by four copper bars (3) and communicate through universal serial ports. Both of them host a 32-bit, 72MHz ARM microprocessor (STM32F1 series). The top sensor board manages the overall logic computation and communicates wirelessly with the server computer using the radio module through the customized communication protocol (see Supplementary Note 6 for detailed information). The bottom actuation board is for the robot motion control. Each robot is equipped with two 3D-printing wheels (4) actuated by the step motor (5), which can drive the robot with the maximum speed of 25 $\mathrm{mm\,s^{-1}}$ and the maximum turning angle of 0.83 $\mathrm{rad\,s^{-1}}$ (see Supplementary Note 6 for detailed information). A universal wheel (7) is mounted at the bottom to keep the robot standing upright. Two 3.7V rechargeable batteries (8) provided energy for about 1 hour in our experimental settings. The passive infrared reflective balls (9) for the NOKOV motion capture system are mounted on the elliptical deck (10). The main and minor axis of the deck are 150mm and 80mm, respectively. b, The experimental system is able to support 10^2 magnitudes of miniature robots. Due to the constraints of the experimental arena, a maximum of 50 robots were used in the collective turn experiments. c, the swarm robotics validation system (i.e., SwarmBang system) comprises three primary components: firstly, a server computer; secondly, a motion capture system; and thirdly, a large number of swarm robots. The server computer, equipped with a radio transmitter, is tasked with model computing, simulating local perception, and transmitting real-time control commands to the robots. The motion capture system is used to locate the position of each robot.

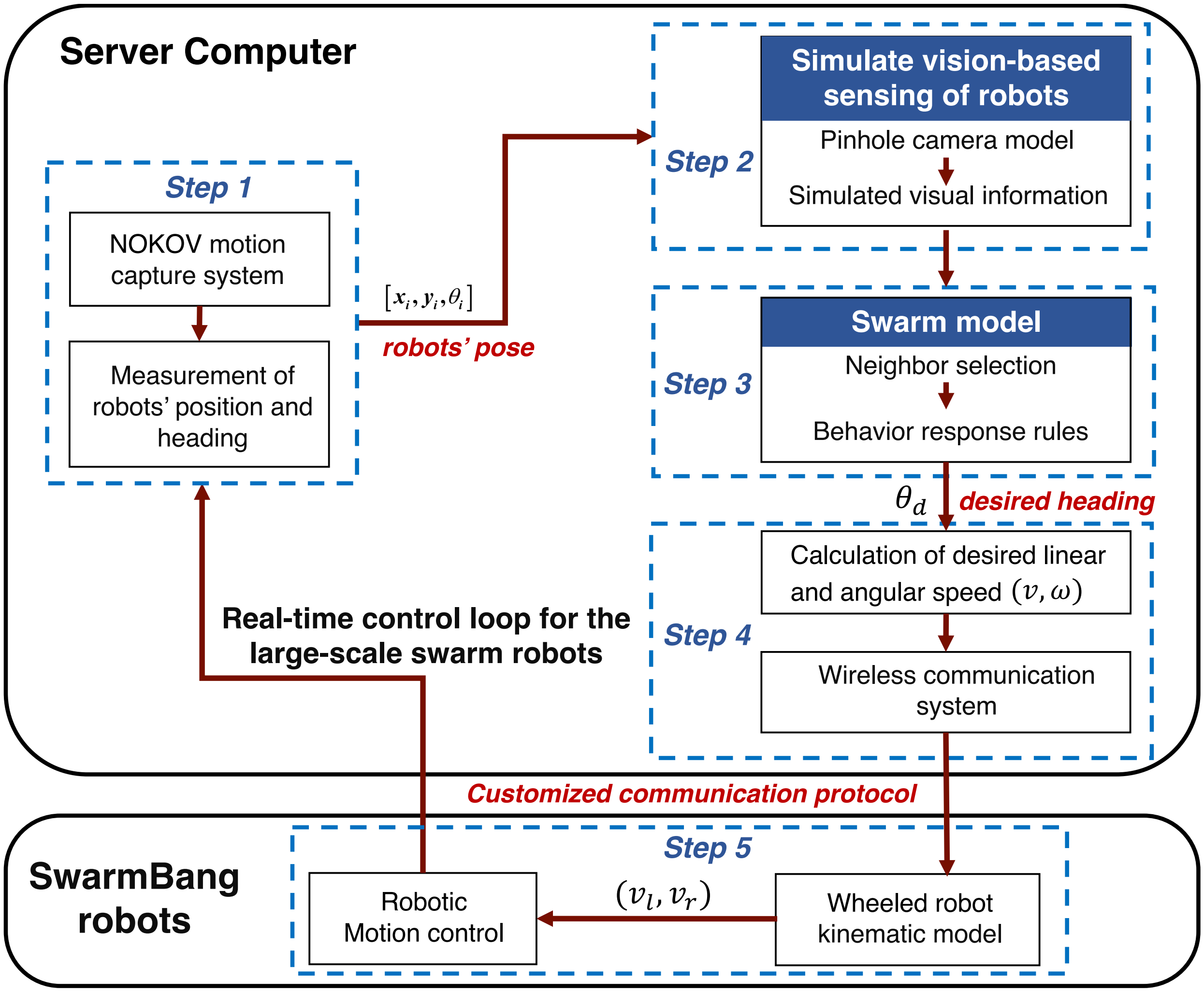

Supplementary Figure 17 | The workflow of the swarm robotic validation system. The workflow of our validation system begins with the measurement of each SwarmBang robot's position and heading using the NOKOV motion capture system processed on the server computer (step 1). Next, the server computer then simulates the local vision-based sensing of each robot through the pinhole camera model (step 2). Then, the server computer determines the desired heading θ_d for each robot based on the corresponding swarm model, utilizing the local visual perception information (step 3). After obtaining the desired heading of each robot, the desired angular speed ω is calculated as the $\omega = \min\left(\frac{\Delta\theta}{\Delta t}, \omega_{\max}^{\text{robot}}\right)$, where the $\Delta\theta = \theta_d(t) - \theta(t)$ and $\omega_{\max}^{\text{robot}}$ is the maximum angular speed in robotic experiments (see Supplementary Table 3 for parameters selection of robotic experiments). Following that, the central computer calculates the desired linear and angular velocity of each robot and broadcasts to all robots based on a wireless communication module through the customized communication protocol with a fixed time interval Δt (step 4). Upon receiving these desired velocities, each robot then calculates the speed of its left and right wheels based on the kinematic model of the differential-drive platform in the robots (step 5).

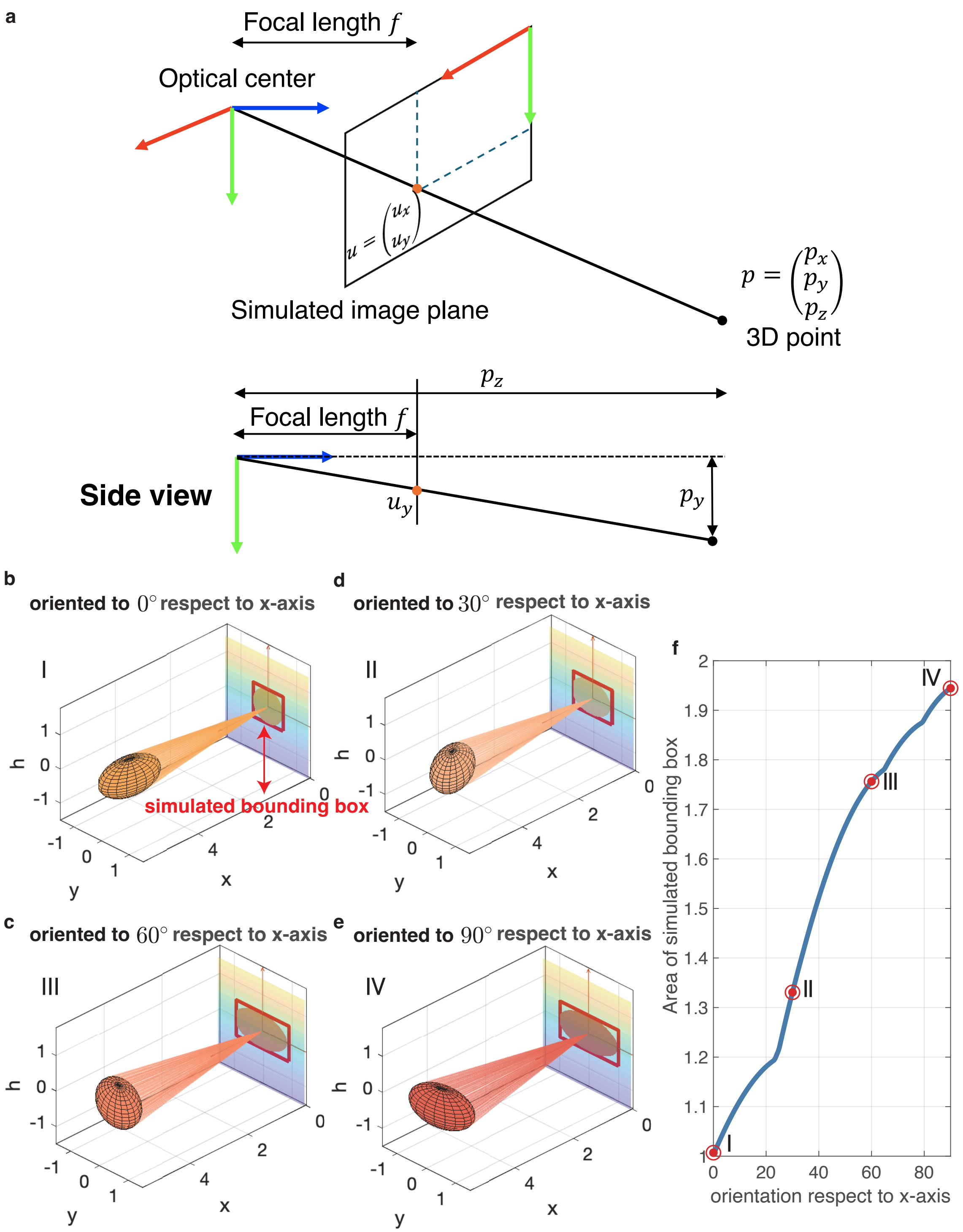

Supplementary Figure 18 | Simulate the vision sensing of the robot through the pinhole camera model. a, the pinhole camera model describes the mathematical relationship between the coordinates of a point in three-dimensional space and its projection onto the image plane. b-e, Using the pinhole camera model, we can obtain the projections of robots (ellipsoids) onto the simulated camera's image plane, where the shape of this projection varies as the robot's orientation changes. To approximate the magnitude of body orientation change from the simulated images, we use the rectangle formed by the maximum X and Y ranges of these projections as the bounding box and quantify the BOC by the variation in the area of bounding boxes (red rectangle in b-e). f, The relationship between the robot's orientation and the corresponding area of the simulated bounding box. We found that as the robot's orientation continuously changes, the area of the bounding box on the simulated image plane also grows steadily, which provides evidence that the variation in the area of the simulated bounding box is practical to estimate the BOC of neighboring robots.

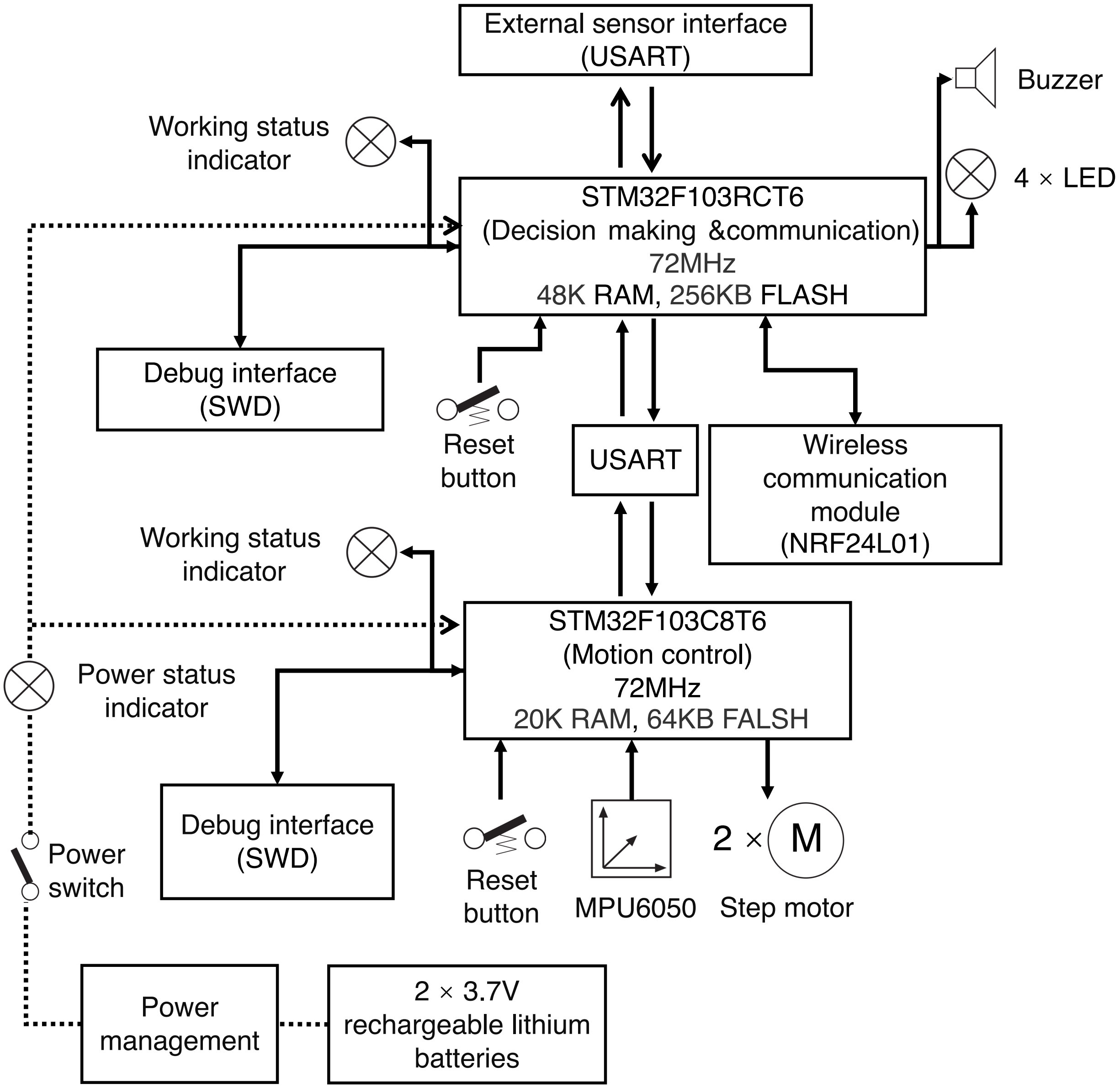

Supplementary Figure 19 | The schematic of SwarmBang robot. The hardware architecture of the SwarmBang robot is separated into two parts: 1) the PCB board for decision-making and communications; 2) the PCB board for motion control and battery management. Two 3.7V rechargeable batteries (2*800mAh) provide energy for about 1 hour in our experimental settings. Each robot is equipped with a wireless communication module (NRF24L01) to receive commands from the server computer.

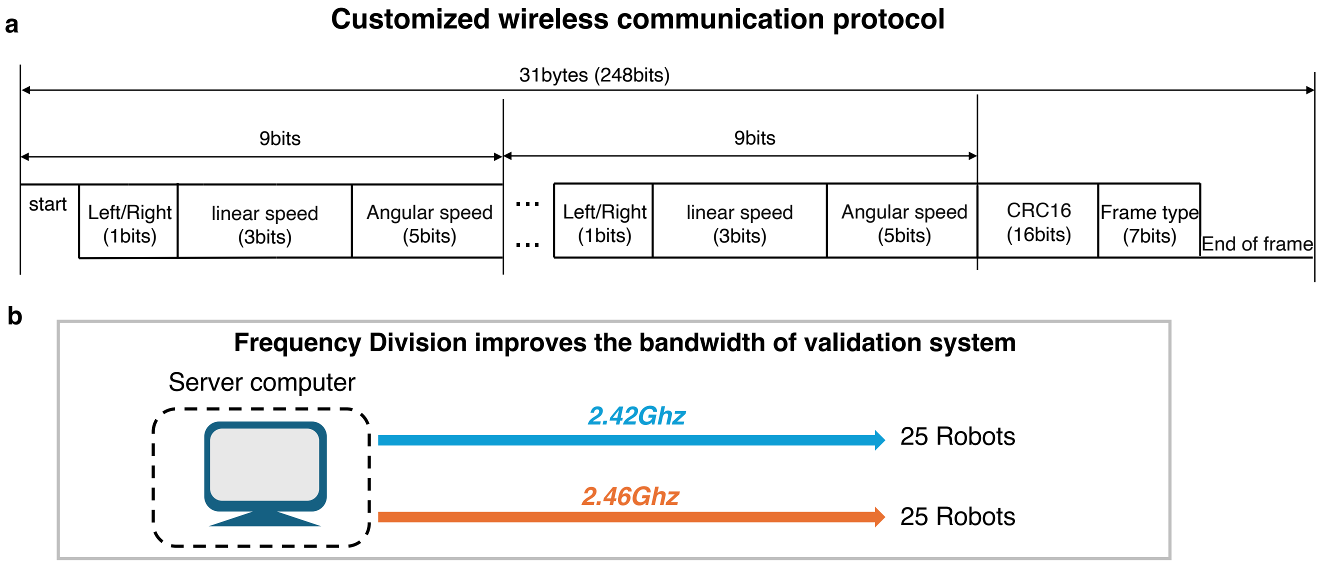

Supplementary Figure 20 | The division of transmission frequency in the wireless communication system and customized wireless communication protocol. a, transmitting motion commands for 50 robots with low communication bandwidth and tight latency requirements poses significant challenges for the robotic validation system. Hence, we developed a low-redundancy communication protocol and employed multiple communication frequencies to simultaneously transmit motion commands, with each frequency corresponding to 25 robots. Each robot's desired linear and angular speeds are represented using 9 bits: 1 bit for indicating the turning direction (0 for left, 1 for right), 3 bits for the desired linear speed, and 5 bits for the angular speed. Each data frame contains the desired linear and angular speeds for 25 robots, totaling $9 \times 25 = 225$ bits. Additionally, we have implemented a Cyclic Redundancy Check (CRC) in each robot to verify the correctness of the received data. Upon receiving a data frame, each robot locates its desired linear and angular speeds within the data frame based on its unique robot ID. b, in our robotic experiment, we employed frequency division multiplexing (2.42GHz and 2.46GHz) to separately control two groups of robots based on the communication protocol, each consisting of 25 robots, totaling 50 robots.

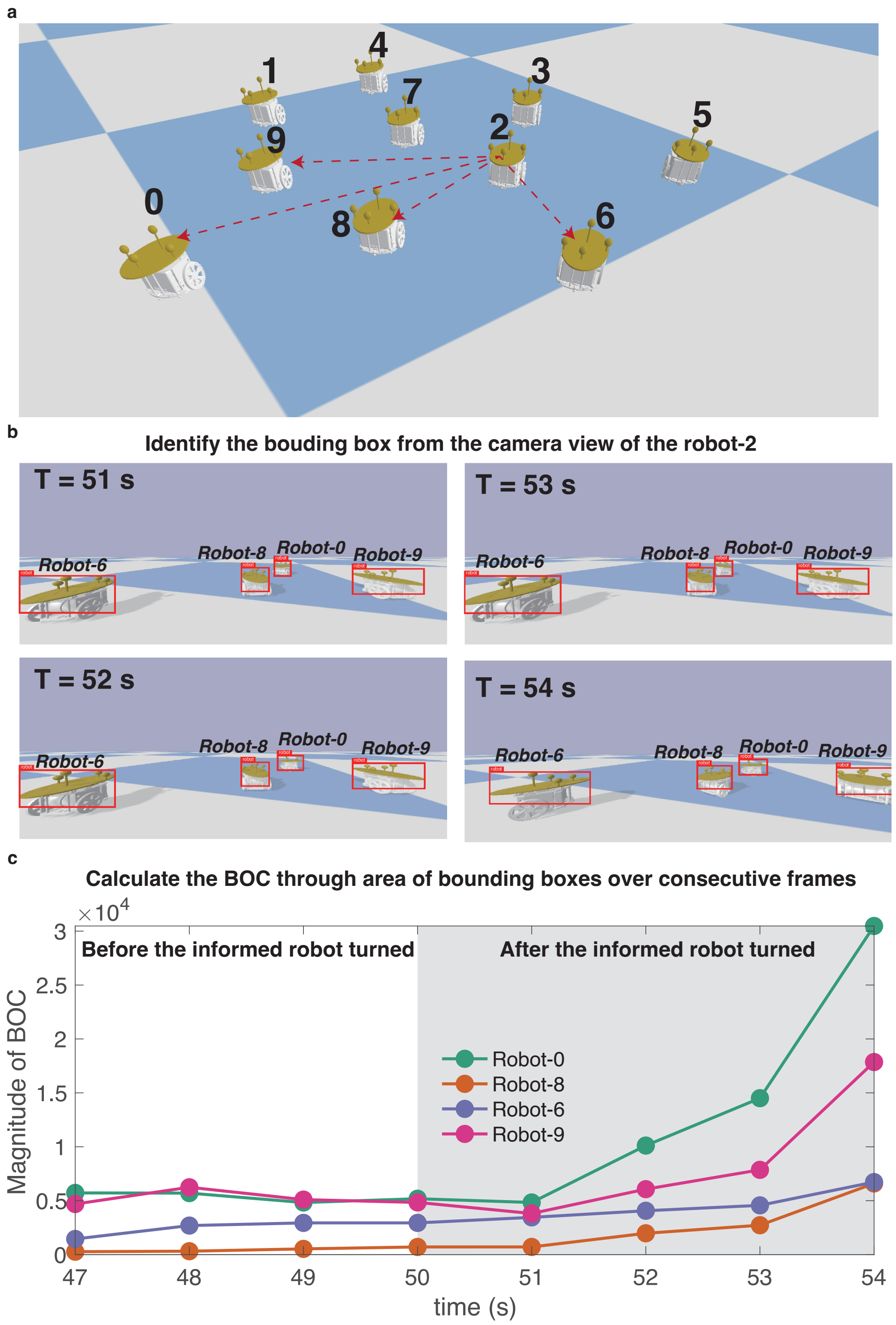

Supplementary Figure 21 | Estiamtion of the BOC using bounding boxes over consecutive frames based on the simulated RGB camera in the pybullet simulator. a, we conducted the simulation experiments of collective turn with a group size of 10 to get the consecutive simulated RGB images from a certain robot's view. The informed individual is the robot-0, which is positioned at the forefront of the group. b, the robot-2 observed four robots in total (robots 0, 8, 6, and 9) and obtained the bounding boxes of the four robots detected from the frames $T = 51s$ to $T = 54s$. c, we estimated the BOC based on the area of the bounding boxes over 2s consecutive simulated image frames. we found that before the informed individual made the abrupt turn, the estimated BOC remained stable for all the perceived robots (robots 0, 8, 6, and 9). After the informed individual suddenly turned, the BOC of robot-0 (the informed individual) is the first to increase, followed by a rising trend in the BOC of the other three robots, indicating that changes in the bounding box area can be used to quantitatively reflect the BOC. The unit of magnitude of BOC calculated from the bounding box is pixel^2.

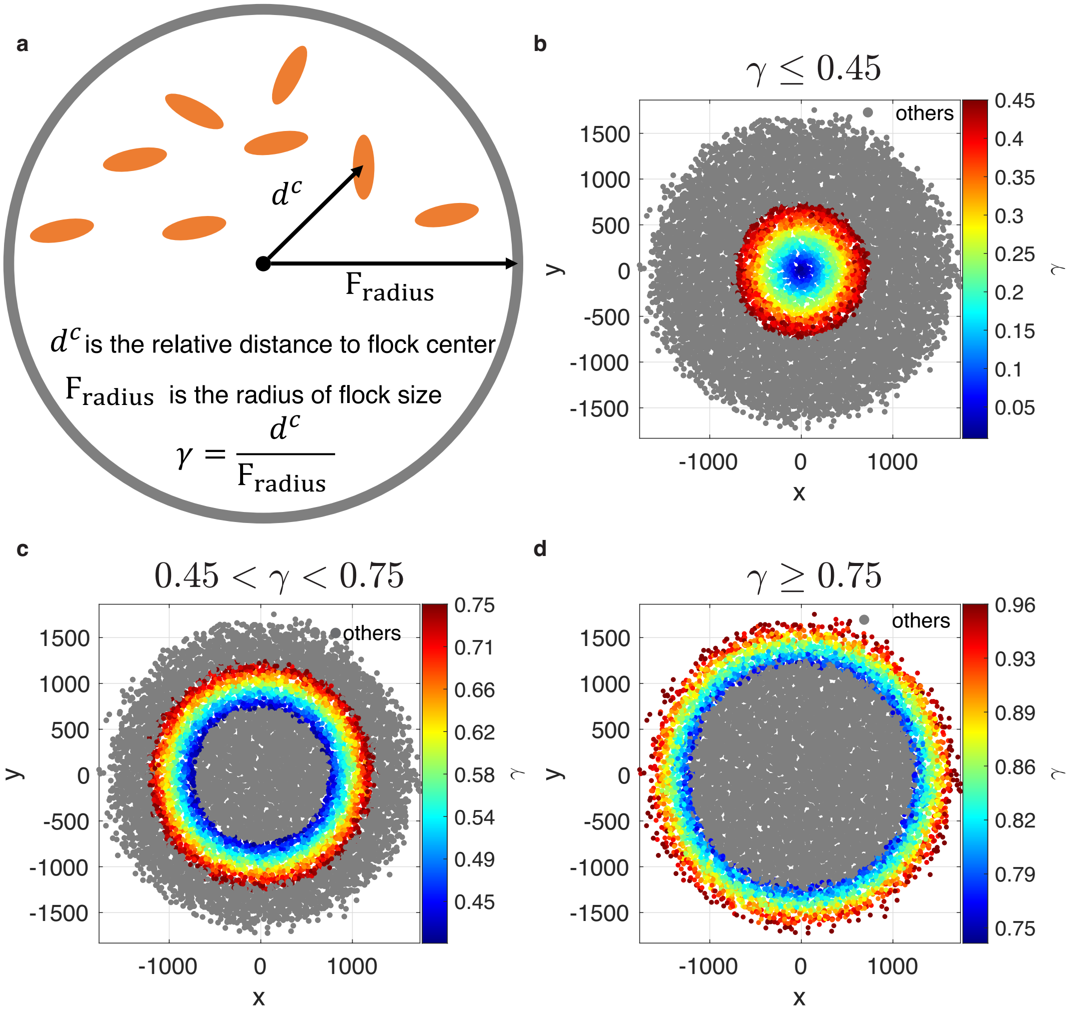

Supplementary Figure 22 | The geometric definition of spatial center proximity index. a, the spatial center proximity index γ is the ratio of the relative distance between the initiator and the center to the radius of the flock, calculated as the $\gamma = \frac{d^c}{\mathrm{F_{radius}}}$. d_c is the relative distance between the initiator and the center. $\mathrm{F_{radius}}$ is the radius of the flock, estimated by half of the maximal relative distance among individuals. b-d, we demonstrate the spatial distribution of individuals with different γ. When the $\gamma \leq 0.45$, the initiator is positioned around the flock center (b). When the $0.45 < \gamma < 0.75$, the initiator is positioned around the middle of the flock (c). When the $\gamma \geq 0.75$, the initiator is positioned around the border of the flock (d).

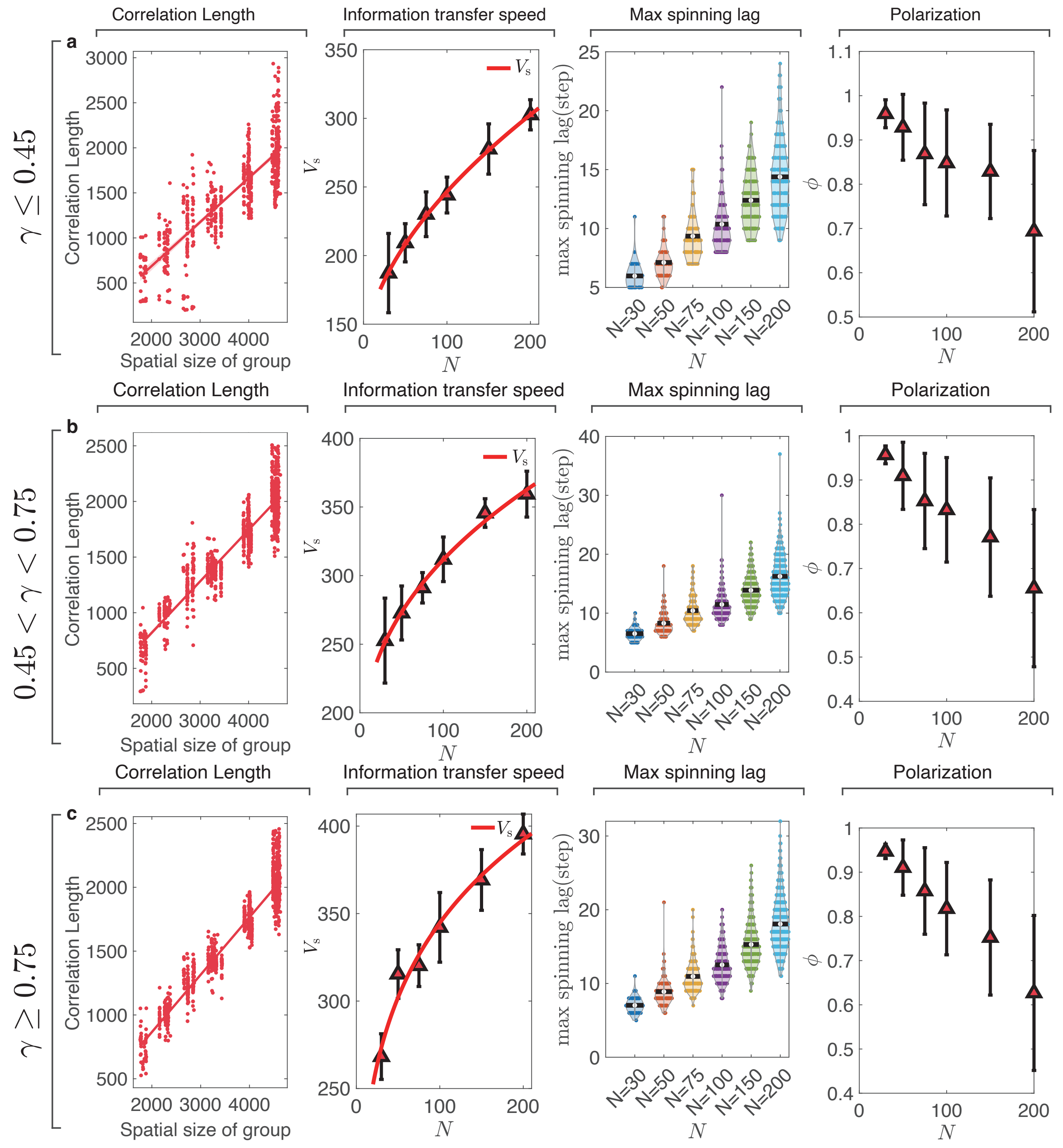

Supplementary Figure 23 | The experiments of collective spin with different positions of informed individuals. We analyzed the impact of informed individual position on information propagation in the experiment of collective spin from four perspectives: the emergence of scale-free correlation, changes in information transfer speed, the maximum spinning lag, and group polarization. As shown in a-c, we found that the position of initiators has negligible impact on the simulation outcome of collective spin. Specifically, the correlation length linearly increases with the flock size when the initiator is positioned around the center, middle and border of the group, indicating the emergence of scale-free correlation. In addition, with the increasing group size, the information transfer speed and the max spinning lag also grow and the group polarization shows the decreasing tendency, which is consistent with the results demonstrated in the main text Fig.3. The unit of information transfer speed is $\mathrm{mm\,s^{-1}}$. The unit of flock size and correlation length are mm, respectively. The error bar in panels (a-c) represents the standard deviation calculated from 50 times independent simulations.

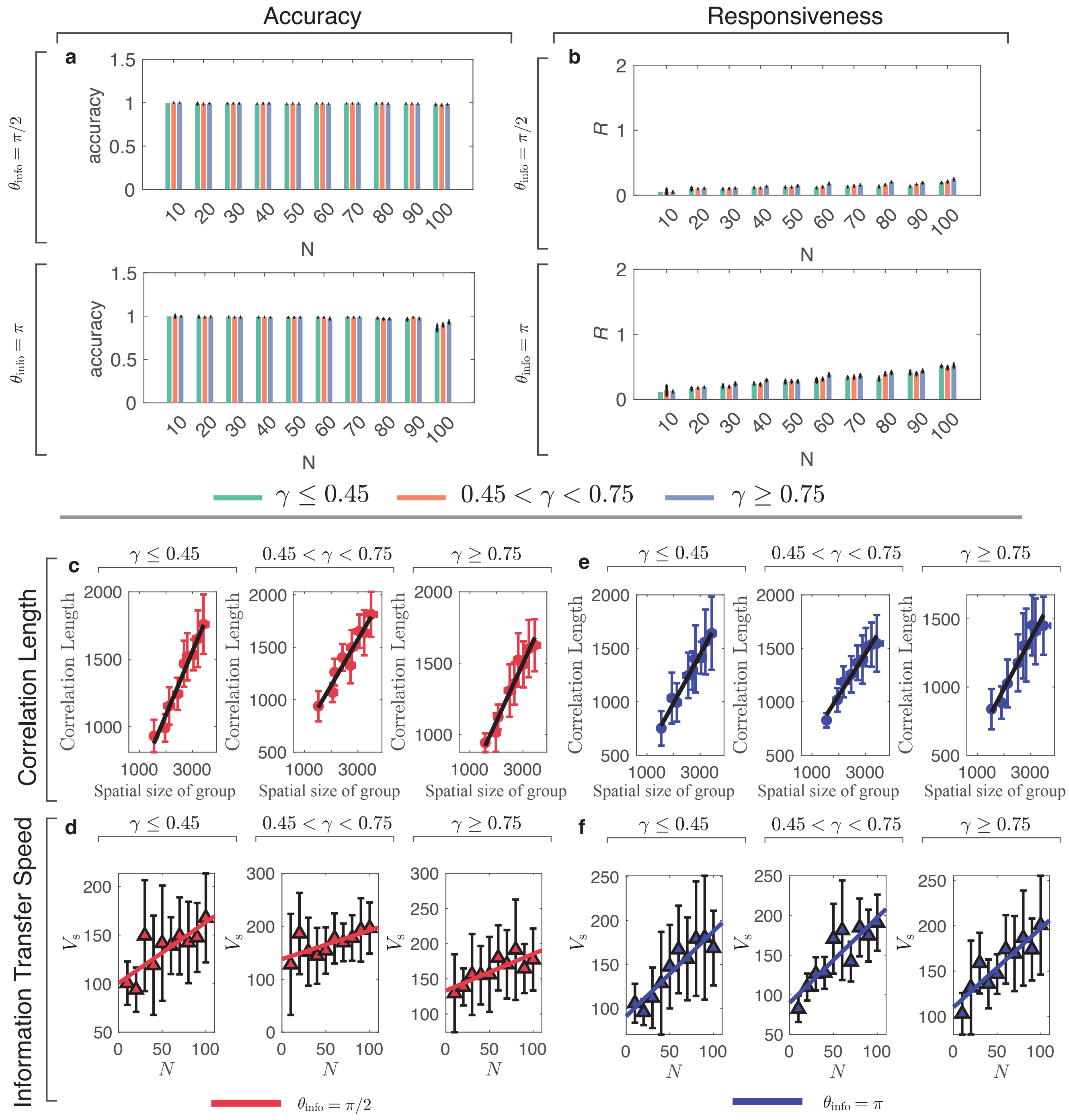

Supplementary Figure 24 | The experiments result of collective turn with different positions of the informed individual. We compare the results of different positions of the informed individual from the response accuracy, responsiveness, change in information transfer speed, and the emergence of scale-free correlation. We found that the simulation outcome of collective turn is barely affected by the initiator's position. No matter whether the initiator is located at the center, middle, or border of the group, the group not only exhibits a high response accuracy in quickly responding to the initiator's sudden turns (a, b) but also demonstrates the emergence of scale-free correlation within the group (c, e). Additionally, the information transfer speed shows an increasing trend with the increase in group size (d, f). These results are consistent with those presented in the main text Fig.4. The unit of information transfer speed is $\mathrm{mm\,s^{-1}}$. The unit of flock size and correlation length are mm, respectively. The error bar in panels (a-f) represents the standard deviation calculated from 50 times independent simulations.

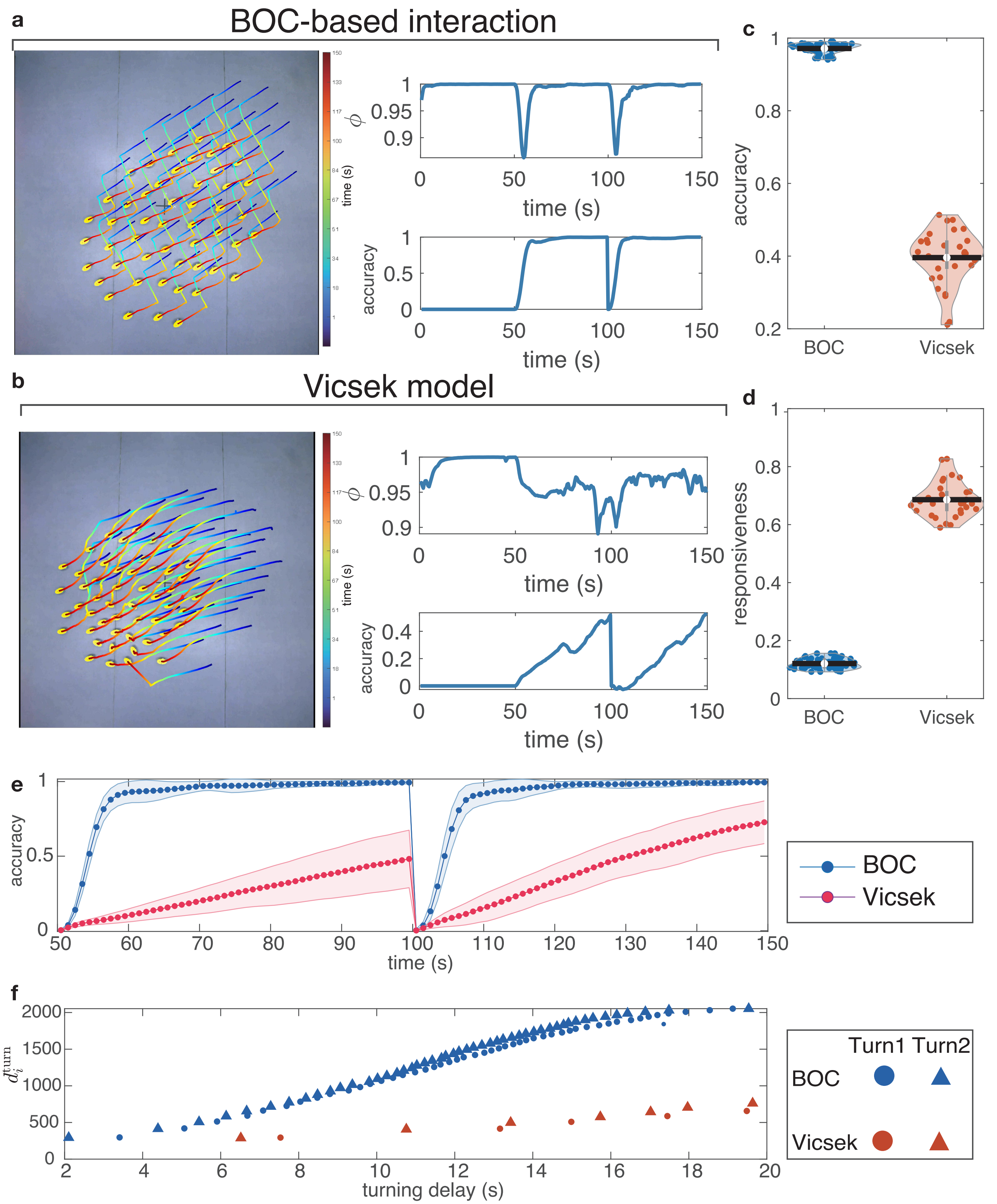

Supplementary Figure 25 | Swarm robotic experiments of BOC-based interaction and Vicsek model. a, The robotic experiment results demonstrate that using BOC-based interaction, the swarm with 50 robots not only successfully follows the informed robot to change its trajectory but also quickly responds to the heading change of the informed robot. b, As a comparison, the swarm using the Vicsek model is difficult to follow the informed robot. c-d, There are noticeable differences in accuracy and responsiveness between BOC-based interaction and the Vicsek model. BOC-based interaction outperforms the Vicsek model in terms of response accuracy and responsiveness. e, The response accuracy as a function of time for the BOC-based interaction and the Vicsek model. The shaded error bar represents the standard deviation. f, The information transfer speed V_s of BOC is much faster than that of the Vicsek model.

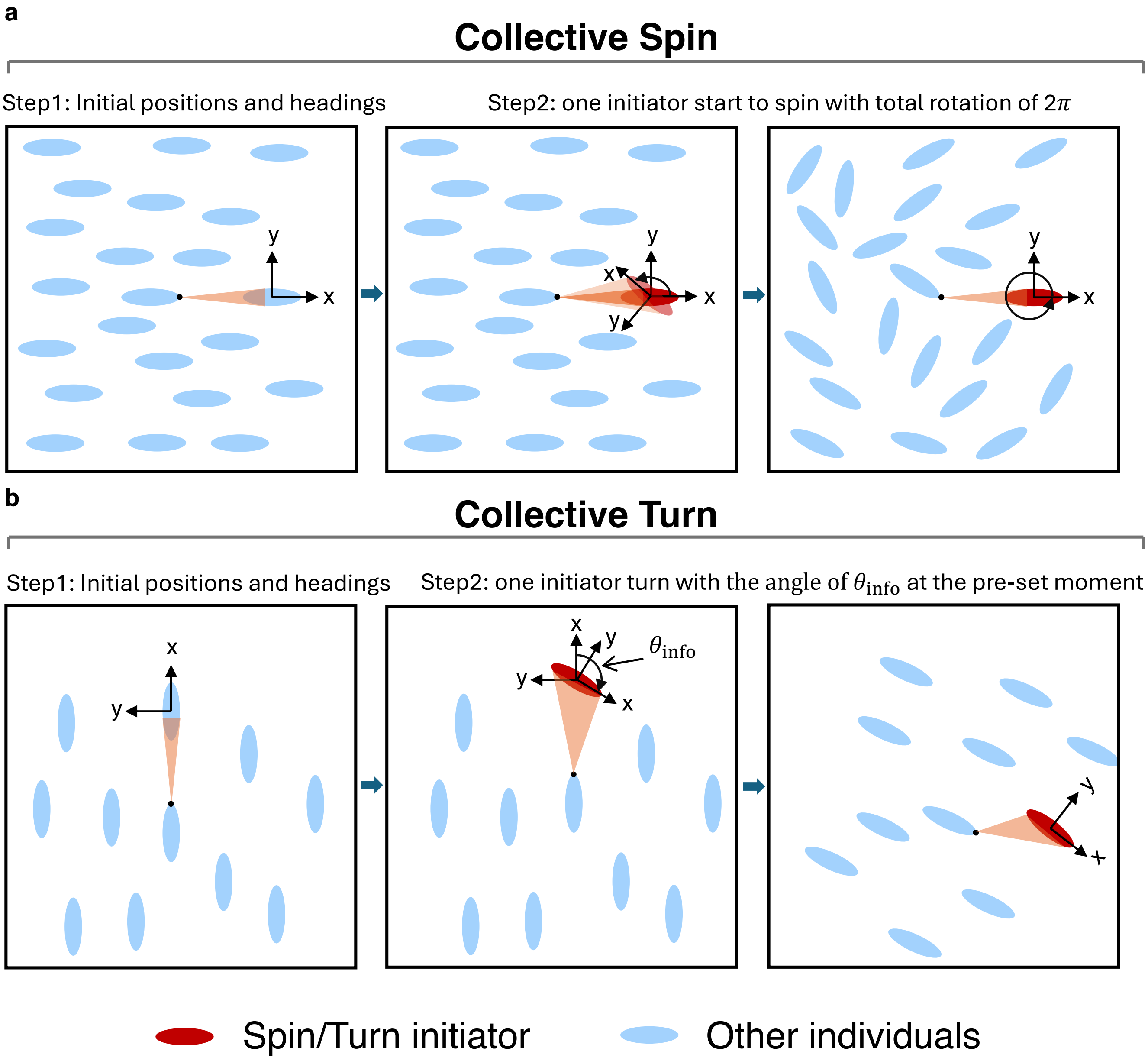

Supplementary Figure 26 | The illustration of simulation experiments of collective spin and collective turn. a, In the simulation experiments of collective spin, we assume that each simulated robot has two motion states: one is stationary, and the other is spinning. The stationary state means that both the linear and angular speed of the simulated robot are set to 0. The spinning state means that the angular speed is set to the maximum while the linear speed is set to 0, indicating the rotational movement of robots. The simulated robots are required to swiftly transfer the spinning state triggered by a single informed individual (spinning initiator) throughout the entire group. The informed individual starts spinning at a pre-set activation time and stops spinning after completing a 2π rotation, which is selected at the forefront of the group. Each simulated robot calculates the BOC of its neighbors within a perception range R_{visual} and selects the neighbor with the maximum BOC to react, i.e., the BOC-based interaction. The reaction rule in the collective spin has the simplest manner for the focal individual, which is to switch between stationary and spinning states based on the state of the selected neighbor. Specifically, if the selected neighbor is in a spinning state, the focal individual also enters the spinning state and starts to spin with the maximal angular speed; if the selected neighbor is stationary, the focal individual switches to the stationary state and stops spinning. b, In the experiments of collective turn, collective turn refers to the coordinated directional changes within the group, where simulated robots are required to respond promptly to a sudden turn initiated by one informed individual (turn initiator). The local interaction is the same as the experiments of collective spin, that each simulated robot calculates the BOC of its neighbors within a perception range R_{visual} and selects the neighbor with the maximum BOC to react. The reaction rule involves the commonly used velocity alignment, i.e., averaging the headings of the selected neighbors and one's own heading (Eq. (7) in the main text). Several assumptions are made to streamline the study of information propagation of BOC-based interaction in the simulation experiments of the collective turn. On the one hand, we assume the informed individual, positioned at the forefront of the group, turns with an angle θ_{info} relative to the group's movement direction at the pre-set moments. On the other hand, due to the challenges in acquiring neighbors' velocity from the first-person perspective, we assume that the focal individual could access the neighbors' velocity information.

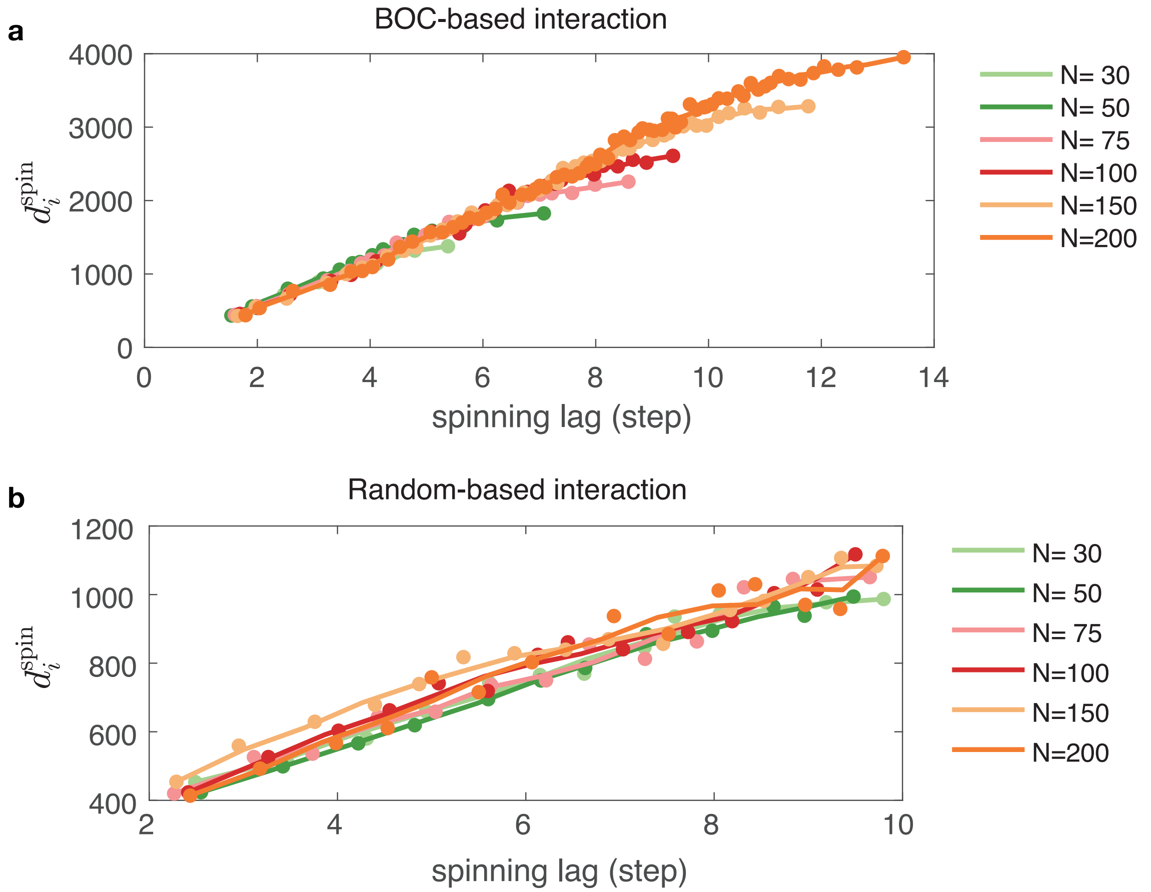

Supplementary Figure 27 | The information transfer distance as a function of spinning lag for BOC and random-based interaction in the simulation experiments of collective spin. a, The slope of curves formed by the information transfer distance and the spinning lag increases with group sizes in the group with BOC-based interaction. b, However, those curves in the group with random-based interaction are almost parallel to each other, and the slope of the curves remains nearly constant.

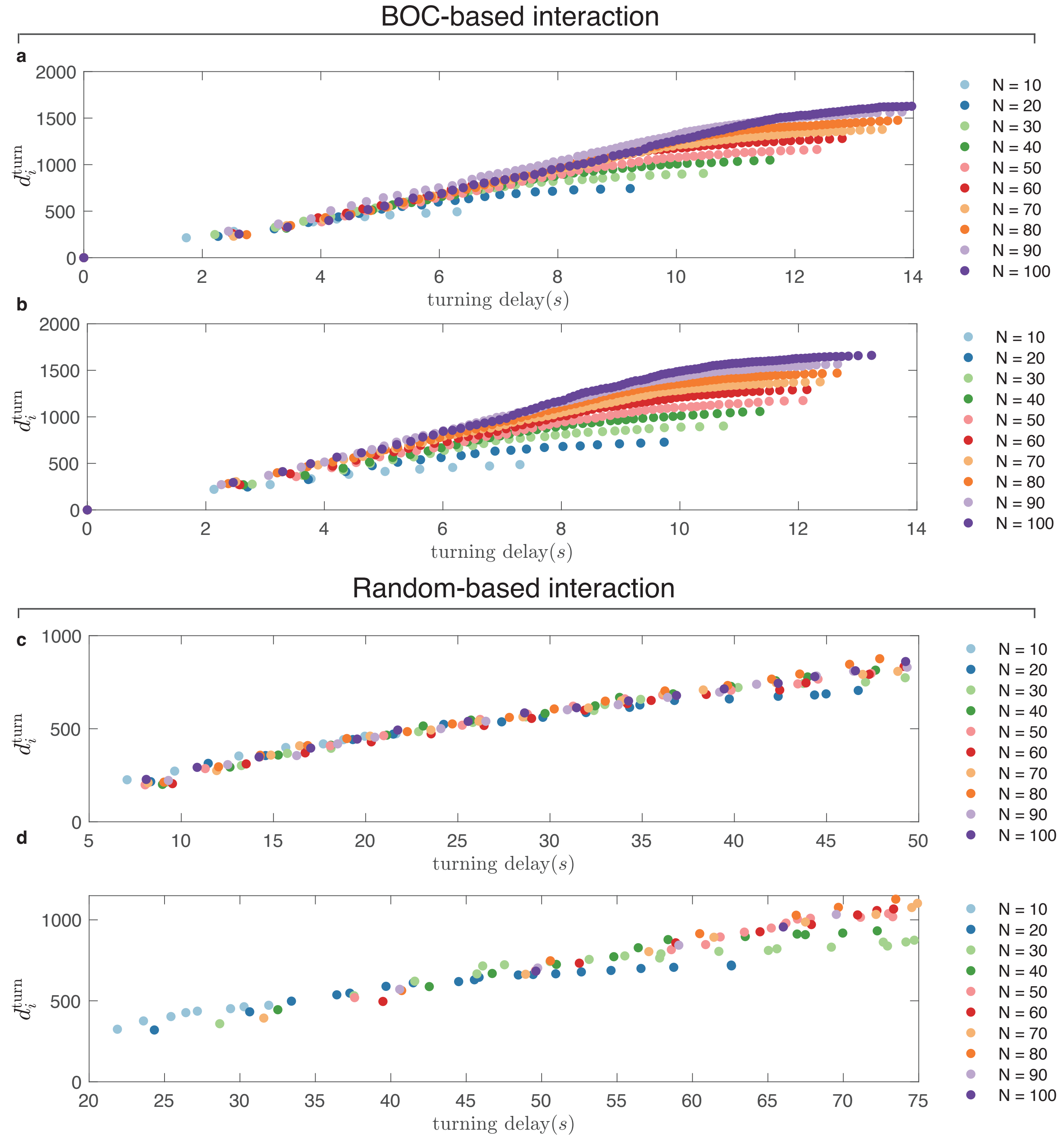

Supplementary Figure 28 | The information transfer distance as a function of turning delay for BOC and random-based interaction in the simulation experiments of collective turn. a-b, The slope of curves formed by the information transfer distance and the turning delay increases with group sizes in the group with BOC-based interaction. c-d, However, those curves in the group with random-based interaction are almost parallel to each other, and the slope of the curves changes slightly.

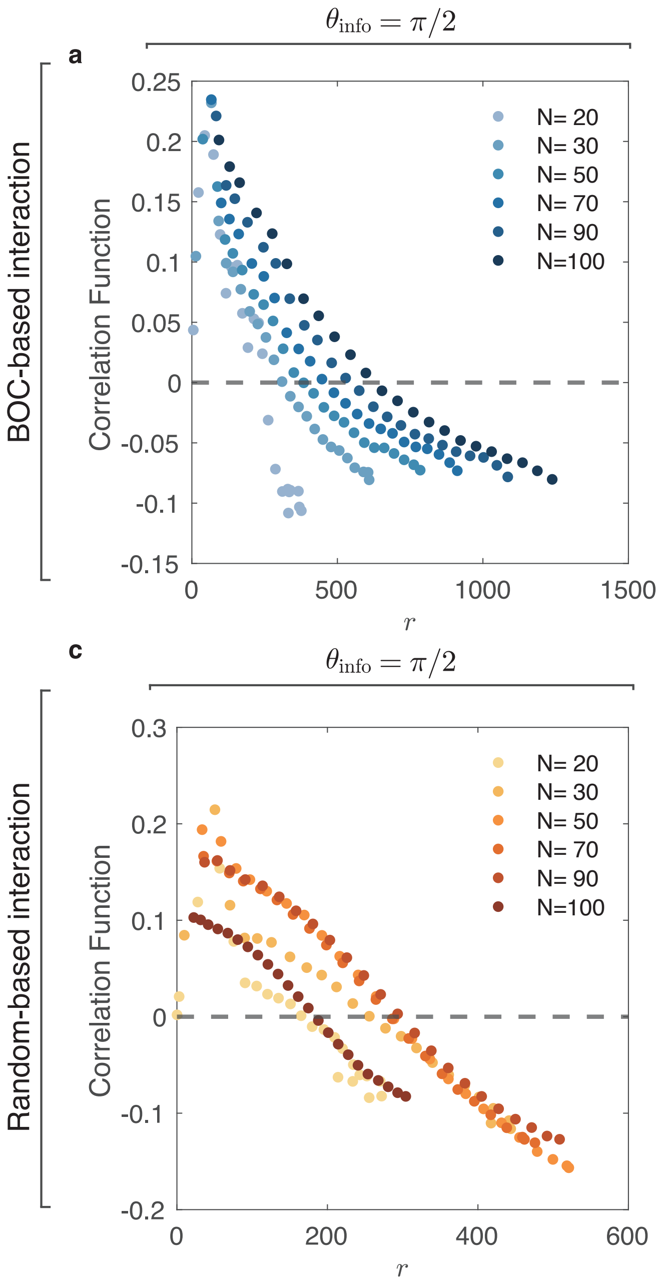

Supplementary Figure 29 | The correlation function as a function of distance for BOC and random-based interaction in the simulation experiments of collective turn. a-b, The point where the correlation function crosses the x-axis increases with the group size, indicating the emergence of scale-free correlation. c-d, The point where the correlation function crosses the x-axis does not change monotonically with increasing group size.

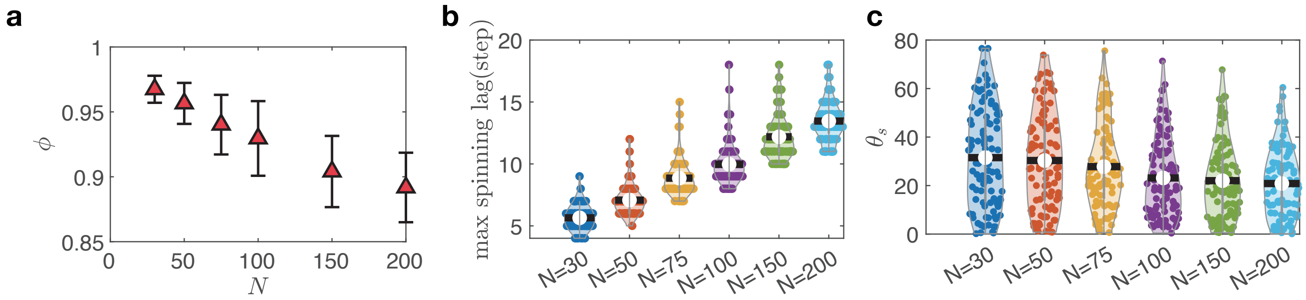

Supplementary Figure 30 | The impact of group size on the BOC-based interaction from the view of group polarization (a), max spinning lag (b), and direction of information transfer (c). The unit of information transfer direction is the degree. The error bar in panel a is the standard deviation.

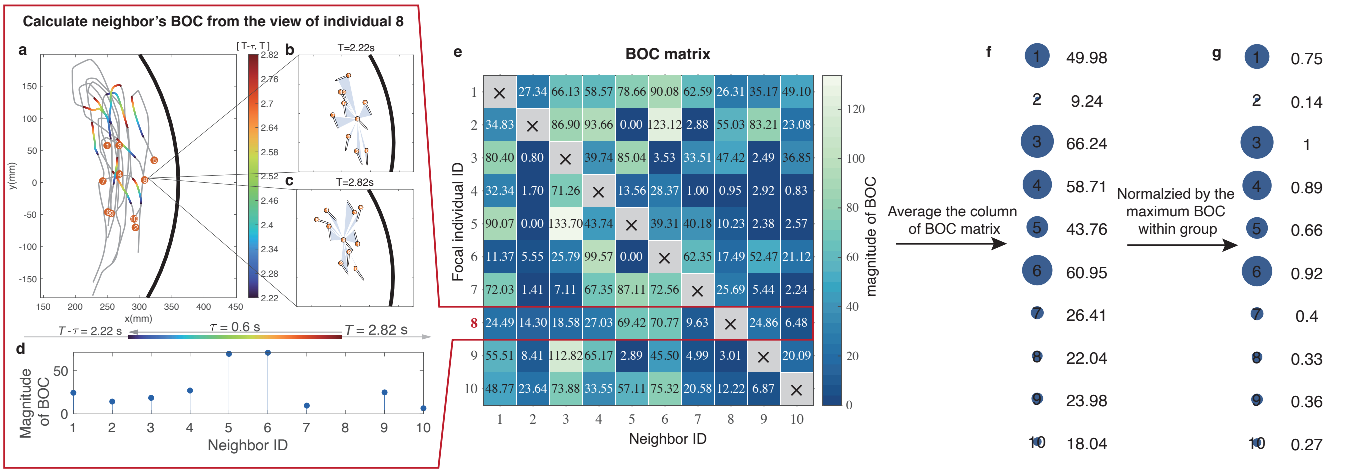

Supplementary Figure 31 | The construction of BOC matrix and derivation of BOC-based motion salience. To get a BOC matrix, we start with the calculation of neighbors' BOC from the view of each individual based on the reconstruction of the visual field. For example, given a period trajectory of U-turn behavior from $T = 2.22s$ to $T = 2.82s$ (a), we reconstructed the visual field of individual 8 (b-c) and calculated the magnitude of its neighbors' BOC based on Eq.(1) in main text (d). As a result, we obtained the eighth column of the BOC matrix (marked by red rectangular shown in e). After obtaining the neighbors' BOC of each individual, we could obtain the complete BOC matrix (e). Then, we calculated the column-wise average to obtain the BOC-based motion salience (f). Additionally, we normalized the BOC-based motion salience by dividing it by its maximum value to ensure that the empirical data analysis is not skewed by differences in the data scales (g). Particularly, the trajectory and BOC matrix presented in panels (a) and (e) are identical to those shown in Fig. 2a-b of the main text.

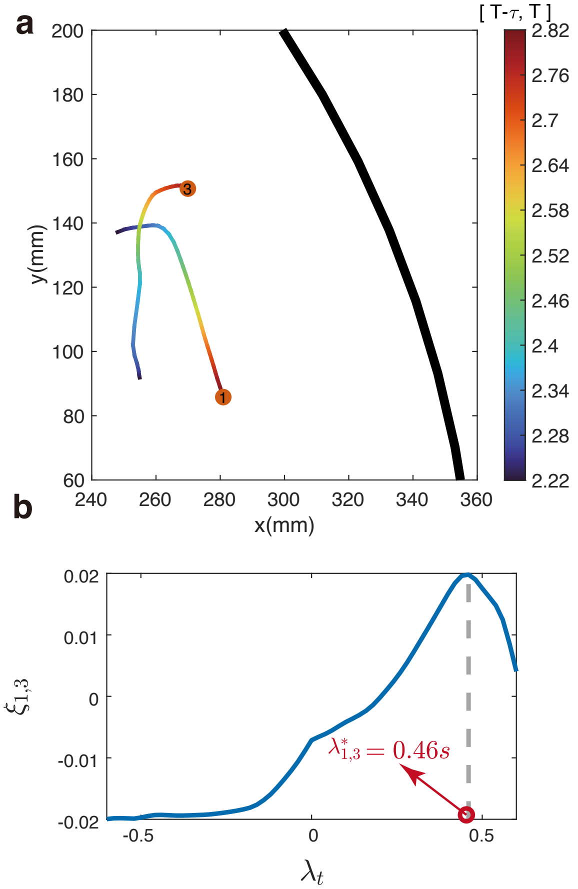

Supplementary Figure 32 | The illustration of determining the relationship between individual 1 and individual 3 based on the sign of maximal time lag λ^*_{ij}. a, The trajectory of individual 1 and individual 3 from $T = 2.22s$ to $T = 2.82s$. b, The curve of directional alignment function $\xi_{1,3}$ with the increasing time lag λ_t, which reaches its maximum at 0.46s. This means that the individual 1 leads the movement of individual 3 by 0.46s ahead.

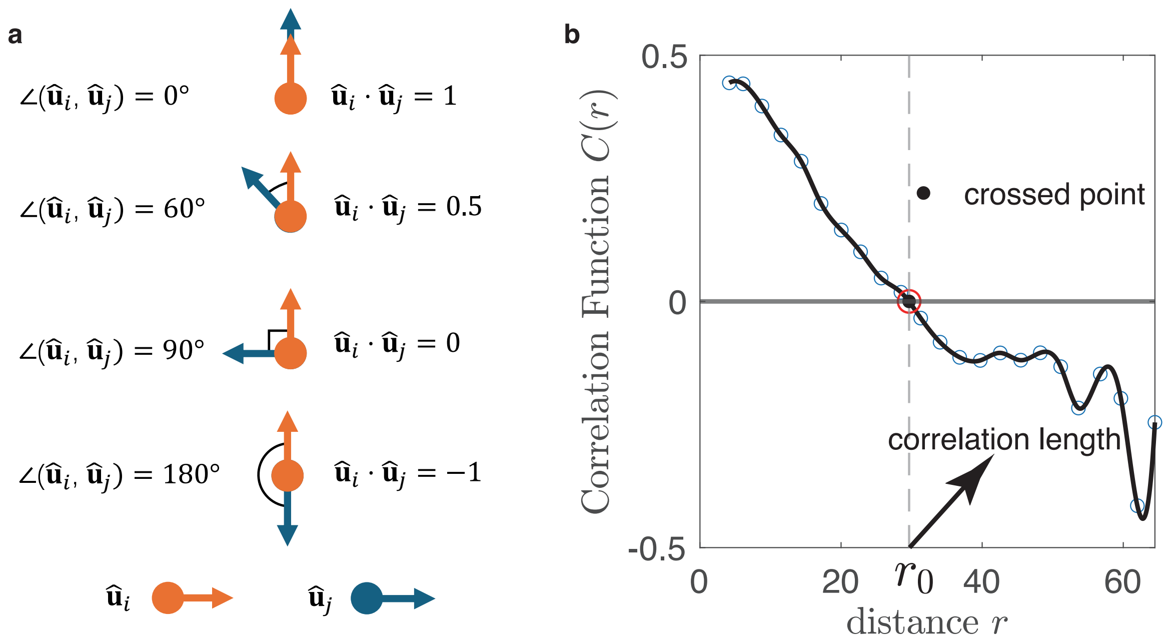

Supplementary Figure 33 | The illustration of the correlation between velocity fluctuation and definition of the correlation length. a, The dot product of velocity fluctuations between individual i and individual j. b, the curve of correlation function $C(r)$ as a function of distance r. The correlation length is defined at $C(r_0) = 0$, which is marked by the red circle.

Supplementary References

References

1. V Lecheval, et al., Social conformity and propagation of information in collective u-turns of fish schools. *Proc. Royal Soc. B: Biol. Sci.* **285**, 20180251 (2018).
2. L Jiang, et al., Identifying influential neighbors in animal flocking. *PLoS computational biology* **13**, e1005822 (2017).
3. E Crosato, et al., Informative and misinformative interactions in a school of fish. *Swarm Intell.* **12**, 283–305 (2018).
4. T Vicsek, A Czirók, E Ben-Jacob, I Cohen, O Shochet, Novel type of phase transition in a system of self-driven particles. *Phys. review letters* **75**, 1226 (1995).
5. CW Reynolds, Flocks, herds and schools: A distributed behavioral model in *Proceedings of the 14th annual conference on Computer graphics and interactive techniques.* pp. 25–34 (1987).
6. JE Herbert-Read, et al., Inferring the rules of interaction of shoaling fish. *Proc. Natl. Acad. Sci.* **108**, 18726–18731 (2011).
7. RP Mann, et al., A model comparison reveals dynamic social information drives the movements of humbug damselfish (dascyllus aruanus). *J. Royal Soc. Interface* **11**, 20130794 (2014).
8. M Beekman, RL Fathke, TD Seeley, How does an informed minority of scouts guide a honeybee swarm as it flies to its new home? *Animal behaviour* **71**, 161–171 (2006).
9. H Ling, et al., Collective turns in jackdaw flocks: kinematics and information transfer. *J. Royal Soc. Interface* **16**, 20190450 (2019).
10. W Poel, C Winklmayr, P Romanczuk, Spatial structure and information transfer in visual networks. *Front. Phys.* **9**, 716576 (2021).
11. H Li, et al., Fast safety distance warning framework for proximity detection based on oriented object detection and pinhole model. *Measurement* **209**, 112509 (2023).
12. AK Fahimipour, et al., Wild animals suppress the spread of socially transmitted misinformation. *Proc. Natl. Acad. Sci.* **120**, e2215428120 (2023).
13. R Harpaz, MN Nguyen, A Bahl, F Engert, Precise visuomotor transformations underlying collective behavior in larval zebrafish. *Nat. communications* **12**, 6578 (2021).
14. F Schilling, F Schiano, D Floreano, Vision-based drone flocking in outdoor environments. *IEEE Robotics Autom. Lett.* **6**, 2954–2961 (2021).
15. A Anoop, P Kanakasabapathy, Review on swarm robotics platforms in *2017 International Conference on Technological Advancements in Power and Energy (TAP Energy).* (IEEE), pp. 1–6 (2017).
16. M Karimi, A Ahmadi, P Kavandi, SS Ghidary, Weemik: A low-cost omnidirectional swarm platform for outreach, research and education in *2016 4th International Conference on Robotics and Mechatronics (ICROM).* (IEEE), pp. 26–31 (2016).
17. J Klingner, A Kanakia, N Farrow, D Reishus, N Correll, A stick-slip omnidirectional power-train for low-cost swarm robotics: Mechanism, calibration, and control in *2014 IEEE/RSJ International Conference on Intelligent Robots and Systems.* (IEEE), pp. 846–851 (2014).
