# Supplementary material for: Body orientation change of neighbors leads to scale-free correlation in collective motion
Source: Nat Commun. 2024 Oct 17;15:8968. doi: 10.1038/s41467-024-53361-8 (PMC11487077; doi:10.1038/s41467-024-53361-8)
Supplement: Supplementary file 2 — Description of Additional Supplementary File [file 41467_2024_53361_MOESM2_ESM.pdf]

Descriptions of Additional Supplementary File

File Name: Supplementary Movie 1
Description: Replay of the typical U-turn behavior of 10 fish. This video showed a replay of the sudden change in direction of 10 fish (i.e., U-turn behavior), with each fish represented as an ellipse, and the head indicated by an orange circle. The U-turn initiator is marked by a red circle.

File Name: Supplementary Movie 2
Description: Replay of the typical U-turn behavior of 8 fish. This video showed a replay of the sudden change in direction of 8 fish (i.e., U-turn behavior), with each fish represented as an ellipse, and the head indicated by an orange circle. The U-turn initiator is marked by a red circle.

File Name: Supplementary Movie 3
Description: The swarm with BOC-based interaction successfully achieves the collective spin in the pybullet simulator. We showed the performance of BOC-based interaction in the collective spin experiment within the Pybullet simulation environment, which shows that a group ($N = 100$) with the BOC-based interaction quickly achieves the collective spin, with all simulated robots in the group executing a rotational movement in place. The spin initiator (or informed individual) is marked by the red circle in this video.

File Name: Supplementary Movie 4
Description: The swarm with random-based interaction failed to achieve the collective spin in the pybullet simulator. This video showed the process of a group ($N = 100$) with random-based interaction in achieving the collective spin. From this video, we can tell the limitations of the random-based interaction in information propagation, preventing the group from achieving the collective spin. The spin initiator (or informed individual) is marked by the red circle in this video.

File Name: Supplementary Movie 5
Description: The swarm with BOC-based interaction successfully responds to the informed robot with turning angle π in the pybullet simulator. This video showed the process of a group ($N = 100$) with the BOC-based interaction achieving a collective turn in Pybullet simulation environment. The turn initiator (or informed individual) turns with $\theta_{\text{info}} = \pi$ and is marked with a red circle after being activated in the simulation.

File Name: Supplementary Movie 6
Description: The swarm with BOC-based interaction successfully responds to the informed robot with turning angle $\frac{\pi}{2}$ in the pybullet simulator. This video showed the process of a group ($N = 100$) with BOC-based interaction achieving a collective turn in the Pybullet simulation environment. The turn initiator (or informed individual) turns with $\theta_{\text{info}} = \frac{\pi}{2}$ and is marked with a red circle after being activated in the simulation.

File Name: Supplementary Movie 7
Description: The swarm with random-based interaction failed to respond to the informed robot with turning angle π in the pybullet simulator. This video showed that a group ($N = 100$) with random-based interaction is unable to achieve a collective turn, as only a subset of individuals can respond to the sudden change in direction of the turn initiator (or informed individual) in the pybullet simulator. The turn initiator (or informed individual) turns with $\theta_{\text{info}} = \pi$ and is marked with a red circle after being activated in the simulation.

File Name: Supplementary Movie 8
Description: The swarm with random-based interaction failed to respond to the informed robot with turning angle $\frac{\pi}{2}$ in the pybullet simulator. This video showcased the failure of a group ($N = 100$) with random-based interaction to respond to the abrupt turn of the initiator (or informed individual) in the pybullet simulator. Even when the turning angle of the informed individual is $\theta_{\text{info}} = \frac{\pi}{2}$, the group is unable to achieve a collective turn. The turn initiator is marked with a red circle after being activated in the simulation.

File Name: Supplementary Movie 9
Description: Robotic experiment of BOC-based interaction with 50 robots. This video records the successful achievement of continuous turns ($\theta_{\text{info}} = \frac{\pi}{2}$) by a swarm of real robots ($N = 50$) with BOC-based interaction. The trajectory of each robot is represented by gray lines, and the initiators of each turn are marked by red circles.

File Name: Supplementary Movie 10
Description: Robotic experiment of random-based interaction with 50 robots. This video showed that a swarm of real robots ($N = 50$) with random-based interaction is unable to achieve any rapid turns initiated by the turn initiator. The group's trajectory

does not exhibit a zigzag movement pattern, but rather an approximately straight line. Each turn initiator (or informed individual) is marked by the red circle.

File Name: Supplementary Movie 11

Description: Robotic experiment of Vicsek model with 50 robots. This video showcased the performance of the Vicsek model during successive collective turns. It is evident that the robotic swarm ($N = 50$) with the Vicsek model is also unable to respond swiftly to the abrupt turn of the initiator (or informed individual), resulting in a failure to achieve continuous collective turns. The turn initiator (or informed individual) is marked by the red circle.
